# Synergistic effects of radiotherapy and immunotherapy: improving oncological outcomes

**DOI:** 10.3389/fimmu.2026.1771355

**Published:** 2026-02-04

**Authors:** Xueqin Chen, Wen Liu, Yuzhu Wang, Zhengliang Yue, Jiajie Wang, Yun Liu, Lifan Xu, Jianjun Hu

**Affiliations:** 1 Department of Dermatology, Southwest Hospital, Army Medical University, Chongqing, China; 2 Institute of Immunological Innovation and Translation, Chongqing Medical University, Chongqing, China; 3 Institute of Immunology, Third Military Medical University, Chongqing, China; 4 Department of Thoracic Surgery, Southwest Hospital, Army Medical University, Chongqing, China; 5 Department of Gastroenterology, Southwest Hospital, Army Medical University, Chongqing, China; 6 Department of Oncology, Southwest Hospital, Army Medical University, Chongqing, China

**Keywords:** artificial intelligence, combination therapy, dose optimization, immunotherapy, radiotherapy, treatment toxicity, tumor microenvironment

## Abstract

Radiotherapy (RT) and immunotherapy, which are cornerstone modalities in the realm of oncology, involve distinct mechanistic pathways and possess unique therapeutic potential. RT achieves localized tumor control by inducing DNA damage and disrupting the tumor microenvironment (TME), whereas immunotherapy—particularly immune checkpoint inhibitors (ICIs)—reactivates dormant antitumor immune responses to exert systemic effects. Across randomized evaluations, evidence for RT–immunotherapy superiority over standard regimens remains inconsistent, with multiple studies failing to show improvement in primary survival endpoints. This result highlights the need for the refined optimization of combinatorial strategies. In this review, we summarize the underlying mechanisms of RT–immunotherapy synergy and actionable strategies to increase therapeutic efficacy. Notably, we elaborate on the dose–immune window hypothesis, which delineates how distinct radiation doses modulate immune responses to achieve synergy with immunotherapy, and we highlight recent advances in artificial intelligence (AI) for optimizing treatment planning, patient stratification, and toxicity predictions. Overall, this review underscores the potential of RT–immunotherapy combinations and provides a framework for precision-based optimization, aiming to guide clinical practice and inspire future research in improving oncological outcomes.

## Introduction

Radiotherapy (RT) and immunotherapy have independently reshaped modern oncology, yet they address different vulnerabilities of cancer. RT exerts cytotoxicity via direct ionizing radiation-induced DNA damage and indirect injury mediated by water radiolysis and reactive oxygen species (ROS), culminating in complex lesions, including single- and double-strand breaks. Beyond tumor cell death, therapeutic irradiation also reprograms the tumor microenvironment (TME) by triggering damage response programs, altering cytokine landscapes, and differentially affecting radiosensitive versus radioresistant stromal and immune subsets, thereby creating immunomodulatory cues (1, 2). However, its clinical effects are largely confined to the irradiated area, leading to a limited systemic impact. Additionally, the application of RT frequently encounters challenges such as radiation resistance, tumor recurrence, and collateral damage to adjacent healthy tissues, which can result in significant toxicity and restrict its efficacy in advanced or metastatic settings (3).

In parallel, immune checkpoint inhibitors (ICIs) targeting cytotoxic T-lymphocyte antigen 4 (CTLA-4) or the PD-1/PD-L1 axis restore antitumor T-cell activity by releasing inhibitory checkpoint signaling and have achieved durable benefits across multiple malignancies, including melanoma, non-small cell lung cancer (NSCLC), and renal cell carcinoma (RCC) (4–6). Nevertheless, primary and acquired resistance remain common, and immune-related adverse events (irAEs)—ranging from mild to life-threatening—can necessitate treatment interruption; toxicity generally increases with dual-checkpoint blockade (6, 7). These realities underscore the need for rational combinations that expand the benefits while mitigating the risk.

The biological rationale for integrating RT with immunotherapy is compelling. RT can endow dying cancer cells with features of immunogenic cell death (ICD), in which effective antigenicity (availability of tumor antigens) and adjuvanticity (spatiotemporally coordinated emission of damage-associated molecular patterns (DAMPs) that recruit and activate mature antigen-presenting cells) cooperate to prime adaptive immunity (8). In preclinical and clinical settings, local irradiation can liberate tumor antigens, promote dendritic cell cross-priming, and convert the irradiated lesion into an *in situ* vaccine, thereby amplifying systemic CD8^+^ T-cell responses—effects that are potentiated by checkpoint blockade (9). Moreover, RT-induced DNA damage responses intersect with immune signaling (for example, the modulation of PD-L1 expression and nucleic acid-sensing pathways), providing additional nodes for synergistic effects (1).

The clinical relevance of RT–immunotherapy combinations is increasingly supported by preclinical and clinical evidence, albeit with variability across diseases. The phase III PACIFIC trial established consolidation durvalumab after concurrent chemoradiotherapy as a standard of care for unresectable stage III NSCLC, with sustained improvements in overall survival and progression-free survival at the 5-year follow-up (10). In addition to NSCLC, multidisciplinary syntheses in melanoma and prospective signals in other solid tumors suggest the feasibility and potential synergistic effects of combination treatments, although the maturity and strength of evidence differ by setting. A multicenter phase II study of IMRT reirradiation with nivolumab reported promising 1-year progression-free survival (PFS) rates for patients with HNSCC over historical expectations with acceptable safety, supporting further evaluation. The PEARL biomarker trial testing preoperative pembrolizumab with short-course RT reported high pCR rates and favorable event-free survival for patients with triple-negative breast cancer (TNBC), along with the dynamic modulation of PD-L1 and tumor-infiltrating lymphocytes (TILs), underscoring biological activity and the importance of sequencing (11–13). The abscopal effect—regression of nonirradiated lesions—remains uncommon but provides a mechanistic insight into systemic immune activation, particularly when RT is integrated with ICIs (14, 15).

Despite these advances, key clinical and mechanistic questions persist. The optimal dose fractionation and sequencing of RT relative to immunotherapy are not yet defined; excessively high single-fraction doses (≥12–18 Gy) can induce three prime repair exonuclease 1 (TREX1) expression and dampen RT-elicited immunogenicity, whereas a field design and lymphocyte exposure can modulate the outcomes, indicating the need for “immune-adaptive” RT planning (11, 16). Moreover, irradiating tumor-draining lymph nodes may compromise systemic T-cell priming and attenuate abscopal responses, suggesting that nodal-sparing strategies should be used when they are oncologically acceptable (17). Treatment sequencing can also shape intratumoral immunity; reduction in TILs after adding RT to anti-PD1 in patients with TNBC, highlighting timing as a determinant of efficacy (13).

In parallel, tumor and patient heterogeneity, including the TME composition and baseline immune competence, require biomarker-guided personalization to increase the number of responders (14). Finally, a combined modality therapy necessitates vigilant toxicity management to balance local radiation injury with immune-related adverse events (16).

In summary, integrating RT with immunotherapy represents an emerging paradigm that aims to unite durable systemic control with reliable local ablation. While early landmark trials (for example, PACIFIC) validated this strategy in defined settings, fully realizing its potential will require mechanistic insights, protocol optimization (dose/fractionation, fields, and sequence), and predictive biomarkers (such as PD-L1 and TILs) to guide patient selection (10, 13, 16). The sections that follow synthesize the underlying biology, clinical applications, and forward-looking solutions to accelerate the safe and effective deployment of RT–immunotherapy combinations.

## Mechanistic insights into radiation therapy-induced immunomodulation

**Figure 1:**
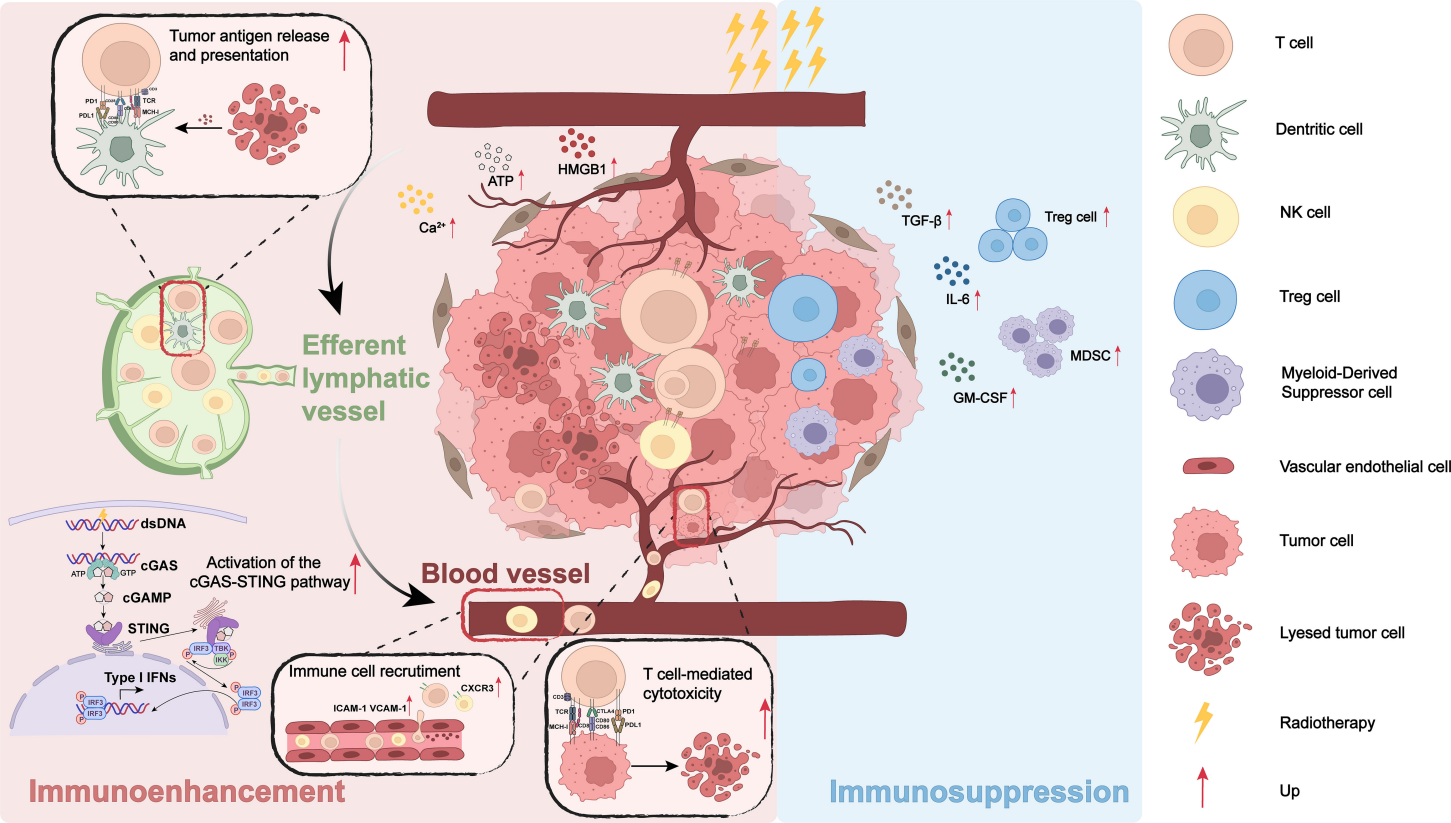
Bidirectional immunomodulation of the tumor microenvironment (TME) by radiotherapy (RT). RT induces immunogenic tumor cell death with DAMP release (ecto-calreticulin, ATP, HMGB1 and Ca²^+^), enhances tumor antigen processing and MHC presentation by dendritic cells, activates the cGAS–STING–IRF3/type-I IFN axis, and upregulates endothelial ICAM-1/VCAM-1 and CXCR3-ligand chemokines to recruit effector T and NK cells, culminating in T-cell–mediated cytotoxicity. Lymphatic trafficking between the irradiated tumor and draining lymph node supports cross-priming and systemic antitumor responses (left, “Immunoenhancement”). Conversely, RT can amplify immunosuppressive programs (right, “Immunosuppression”), including increased TGF-β, IL-6 and GM-CSF, expansion/recruitment of regulatory T cells and myeloid-derived suppressor cells, and adaptive checkpoint up-regulation that constrains effector function. Lightning bolts denote RT; red arrows indicate up-regulation; the legend at right identifies cell types and symbols.

RT is a cornerstone of cancer treatment and is traditionally recognized for its ability to achieve local tumor control through the induction of DNA damage and subsequent apoptosis (18). However, emerging evidence indicates that RT significantly influences the immune system, simultaneously stimulating antitumor immunity and altering immunosuppressive pathways. This dual role emphasizes the necessity of understanding the mechanistic aspects of RT-induced immunomodulation, particularly within the context of combining RT with immunotherapy (19). This section reveals how RT modulates tumor antigen release and presentation triggers immune cell recruitment and activation, and influences immunosuppressive mechanisms (**Figure 1**).

### Radiation-triggered DAMP signaling and antigen presentation dynamics

RT induces ICD, with prototypic DAMPs, including ecto-calreticulin, ATP, and high-mobility group box 1 (HMGB1), serving as “danger signals” that initiate sterile inflammation and promote antigen uptake by antigen-presenting cells (APCs), particularly dendritic cells (DCs). HMGB1, for example, acts as a TLR4-mediated activator that promotes maturation and cross-presentation. In parallel, HMGB1 also signals through TLR2/TLR4 and RAGE on immune cells to drive pro-inflammatory transcriptional programs that support antigen uptake and the priming of cytotoxic T-cell responses (20, 21).

Antigens released from irradiated tumor cells are internalized by DCs and processed for major histocompatibility complex (MHC) class I and class II presentation on APCs, thereby priming CD8^+^ and CD4^+^ T cells, respectively. Concomitantly, RT upregulates MHC class I expression on tumor cells and expands the available intracellular peptide pool, increasing cytotoxic T lymphocytes (CTLs) visibility to irradiated targets. These effects are dose- and time-dependent and mechanistically linked to increased TAP activity and peptide supply. A pivotal bridge between local DNA damage and systemic T-cell priming is cyclic GMP–AMP synthase–stimulator of interferon genes (cGAS–STING)–IRF3 signaling in host DCs. DNA from irradiated tumor cells activates cGAS–STING in DCs, inducing interferon-beta (IFN-β) and other type I interferons (IFN-I)-driven pathways that are required for effective cross-priming and tumor control after RT; exogenous IFN-β can rescue cross-priming in STING- or cGAS-deficient settings (22, 23). In terms of antigenic novelty, RT broadens the antigenic peptide repertoire and can expose radiation-associated neoepitopes/neoantigens recognizable by both CD8^+^ and CD4^+^ T cells; over longer time scales, therapy-induced DNA damage and impaired repair may increase tumor immunogenicity and sensitize tumors to immune checkpoint blockade. These concepts support the view of the irradiated lesion as an *in situ* vaccine, particularly when combined with ICIs (24).

### Immune cell recruitment and activation

In addition to enhancing antigen presentation, RT programs chemokine and endothelial–adhesion cues that govern the directed entry and functional licensing of effector leukocytes (25). RT and downstream interferons upregulate the expression of C-X-C chemokine receptor 3 (CXCR3) ligands (C-X-C chemokine ligand 9 (CXCL9), CXCL10, and CXCL11), which guide CD8^+^ T cells and natural killer (NK) cells into tumors. In preclinical systems, IFN-γ-driven expression of CXCR3 ligands and CXCR3 on effector NK subsets (notably high CD27 expression) are prerequisites for robust NK accumulation; exogenous CXCL10 is sufficient to increase intratumoral NK and T-cell recruitment and prolong survival (26, 27).

In parallel, cGAS–STING signaling in host DCs links local DNA damage to systemic T-cell priming. DNA and micronuclei derived from irradiated tumor cells generate cyclic GMP–AMP (cGAMP), activate STING–IRF3 signaling, and induce the expression of IFN-I, which are required for the effective cross-presentation and priming of tumor-specific CD8^+^ T cells (28). RT also activates and remodels the tumor vasculature, increasing leukocyte extravasation. RT-induced increases in the levels of proinflammatory cytokines (IL-1β, TNF-α, and IFNs) upregulate intercellular adhesion molecule-1 (ICAM-1) and vascular cell adhesion molecule-1 (VCAM-1) expression on the tumor endothelium, thereby facilitating the firm adhesion and transendothelial migration of effector lymphocytes. Complementary pathways, such as CXCL16–CXCR6, can further enhance T-cell homing. In some contexts, RT-triggered ICAM-1/VCAM-1 expression within tumors or the endothelium increases the infiltration and efficacy of cellular immunotherapies, exemplifying how vascular and stromal activation cooperate with chemokine cues (25, 26, 29).

In summary, chemokine-driven recruitment, STING pathway activation, and endothelial cell remodeling create a proinflammatory and immunostimulatory TME that supports robust immune cell infiltration and activation, setting the stage for a coordinated antitumor immune response.

### Immunosuppressive effects

In addition to initiating antitumor immunity, RT can also potentiate immunosuppressive circuits within the TME, a duality that is highly context-, dose-, and fractionation-dependent. Consequently, RT may simultaneously prime antitumor responses and reinforce tolerogenic pathways, necessitating combination strategies that blunt RT-driven suppression while preserving its immunogenic benefits (30).

Regulatory T cells (Tregs). Tregs are central to maintaining immune tolerance and are frequently expanded or functionally augmented after RT. Rather than a simple dichotomy whereby “low-dose RT reduces” and “high-dose RT increases” the number of Tregs, preclinical and translational data indicate that Treg dynamics depend on the tumor context and RT parameters. Notably, high single-fraction doses can skew the TME toward immunosuppression and lymphodepletion, whereas fractionated regimens more often support immunostimulatory effects (31, 32). Mechanistically, RT increases the Treg number and resilience through IL-10/signal transducer and activator of transcription 3 (STAT3) and transforming growth factor-beta (TGF-β)/SMAD signaling, and surviving Tregs can upregulate Akt expression, enhancing radioresistance (19). In glioblastoma, immunosuppressive CD103^+^ Tregs accumulate under radioimmunotherapy pressure and have been implicated in resistance (33).

TGF-β is a principal driver of Treg induction after RT. Latent TGF-β can be activated via integrin-mediated traction and proteolysis or presented on Tregs through GARP/αvβ8, integrating stromal and immune sources of TGF-β activity in irradiated tissues (34). Importantly, Activin A, another TGF-β superfamily member that is upregulated by irradiation, can compensate when TGF-β is blocked; preclinical work has shown that the dual blockade of TGF-β and Activin A is required to prevent RT-induced intratumoral Treg accumulation and to restore CD8^+^ T-cell priming (35). In addition to cytokine cues, chemokines such as C-C chemokine ligand 22 (CCL22) and CCL28 also contribute to Treg recruitment in irradiated tumors (19).

Myeloid-derived suppressor cells (MDSCs). RT-driven tissue injury, hypoxia/vascular changes, and inflammatory mediators can promote MDSC accumulation and activation. Activated MDSCs suppress effector T cells via arginase-1, reactive oxygen species, and nitric oxide, among other pathways; metabolic reprogramming (e.g., enhanced glycolysis) further stabilizes their suppressive phenotype (36, 37). In addition, RT-modulated cytokine and growth factor networks (e.g., IL-1 and VEGFA) support MDSC induction and expansion (19). Therapeutically, MDSC-targeted approaches are being actively evaluated: PDE5 inhibitors (e.g., sildenafil) can blunt MDSC function, ATRA can promote MDSC differentiation and reduce their immunosuppressive function in patients, and the blockade of chemokine signaling (e.g., C-C chemokine receptor type 2 (CCR2)/CCR5) is being combined with hypofractionated RT and PD-1 inhibition in early-phase trials (36).

Another well-recognized immunosuppressive adaptation to RT is the upregulation of PD-L1 expression within the tumor microenvironment. This upregulation occurs not only on cancer cells but also on antigen-presenting and myeloid subsets and is driven by IFN-γ produced by activated CD8^+^ T cells and, in some contexts, by ATR/CHK1-dependent STAT1/3 signaling downstream of radiation-induced DNA damage. As a result, RT-elicited T-cell responses can be curtailed by PD-(L)1 engagement (38). Conversely, this adaptive checkpoint provides a therapeutic target: multiple preclinical models have demonstrated that PD-1/PD-L1 blockade concurrent with RT augments local control, induces durable, CD8^+^ T-cell-dependent immunity, and can reduce intratumoral MDSC accumulation; delayed sequencing is less effective (39).

Taken together, RT exerts bidirectional immune pressure by enhancing antigen presentation, chemokine-guided trafficking, and DC cross-priming while simultaneously inducing the expression of checkpoint ligands, adenosine-generating enzymes, and signaling by TGF-β superfamily members that favor regulatory and suppressive cell states. These insights argue for rational combination designs that shift the balance toward antitumor immunity. In addition to PD-(L)1 inhibitors, priorities include the blockade of the TGF-β/Activin A axis and CD73–adenosine signaling, as well as STING pathway agonism to amplify IFN-I programs; critically, dose fractionation and concurrent scheduling should be optimized to preserve innate sensing and limit adaptive resistance (40). Understanding these mechanisms is essential for optimizing combination regimens and unlocking the full potential of RT-induced immunomodulation in oncology.

Building on this mechanistic framework, the subsequent sections synthesize information from clinical trials of selected diseases in which RT–immunotherapy combinations have been explored, highlighting how the regimen selection and biomarker-guided strategies may translate immune-permissive remodeling into meaningful benefits for patients.

## Clinical applications and case studies

### Key clinical trials and outcomes

The combination of RT and ICIs has been extensively evaluated in clinical trials and has shown significant potential across various types of cancer. These trials have provided critical insights into the efficacy, safety, and mechanistic basis of RT–ICI combinations. Below, we summarize key clinical trials that highlight the progress and challenges in this field (**Table 1**).

**Table 1:** Key clinical trials of RT-ICI integration.

Trials	Publication time	Trial phase	Inclusion criteria	Number of patients	Intervention	End points	Outcome	Safety	TME-related prognosis	Evidence level (OCEBM)
Kwon et al. (41) (CA184–043 trial)	2014	III	metastatic castration-resistant prostate cancer	799	radiotherapy followed by ipilimumab vs. radiotherapy followed by a placebo	OS	Median overall survival: 11.2 months versus 10.0 months	Grade 3 or 4 irAEs: 26% versus 3%	not mentioned	1b
Antonia et al. (42) (Pacific trial)	2017	III	stage III, unresectable NSCLC	713	sequential chemoradiotherapy followed by durvalumab vs. sequential chemoradiotherapy followed by a placebo	PFS	Median PFS: 16.8 months versus 5.6 months	Grade 3 or 4 TRAEs: 29.9% versus 26.1%	A PFS advantage with durvalumab was observed irrespective of pretreatment tumor PD-L1 expression.	1b
Kelly et al. (43) (Checkmate 577 trial)	2021	III	esophageal or gastroesophageal junction cancer	794	chemoradiotherapy followed by nivolumab vs. chemoradiotherapy followed by a placebo	DFS	Median DFS: 22.4 months versus 11.0 months	Grade 3 or 4 TRAEs: 13% versus 6%	A DFS benefit was observed in the nivolumab group, regardless of PD-L1 expression.	1b
Siva et al. (44) (the RAPPORT trial)	2022	I/II	oligometastatic clear cell renal cell carcinoma	30	stereotactic radiotherapy followed by short-course pembrolizumab	DCR, ORR, PFS, OS	DCR: 83% ORR: 63% PFS: 60% (1 year), 45% (2 years) OS: 90% (1 year), 74% (2 years)	Grade 3 TRAEs: 13%; no grade 4 or 5 AEs	not mentioned	4
Chen et al. (45) (CheckPAC trial)	2022	II	refractory metastatic pancreatic cancer	84	SBRT followed by nivolumab vs. SBRT followed by nivolumab and ipilimumab	CBR	CBR: 17.1% vs. 37.2%	Grade 3 or higher TRAEs: 24.4% vs. 30.2%	The expression of PD-L1 was not associated with a clinical benefit,	1b
Kwan et al. (50) (ICE-PAC trial)	2022	II	metastatic castration-resistant prostate cancer	31	stereotactic ablative body radiotherapy followed by avelumab	DCR, ORR, rPFS, OS	DCR: 48% ORR: 31% median rPFS: 8.4 months median OS: 14.1 months	Grade 3 or 4 TRAEs: 16%	not mentioned	2b
Tachihara et al. (46) (DOLPHIN trial)	2023	II	locally advanced NSCLC	35	radiotherapy in combination with concurrent and maintenance durvalumab	12-month PFS	12-month PFS: 72.1%	Grade 3 or 4 AEs: 52.9%; Grade 5 AEs: 5.9%	Patients with a PD-L1 TPS higher than 50% experienced longer PFS than those with a value lower than 50%.	2b
Chiang et al. (51) (START-FIT trial)	2023	II	locally advanced, unresectable hepatocellular carcinoma	33	TACE followed by radiotherapy followed by avelumab	proportion of patients deemed amenable to curative treatment	18 (55%)	Grade 3 or worse TRAEs: 33%	Patients with a higher PD-L1 concentration (>250 pg/ml) had a higher complete response rate.	2b
Lorusso et al. (47) (KEYNOTE-A18 trial)	2024	III	newly diagnosed, high-risk, locally advanced cervical cancer	1060	chemoradiotherapy plus pembrolizumab vs. chemoradiotherapy plus a placebo	PFS and OS	Median PFS: rates at 24 months were 68% versus 57% OS: rates at 24 months were 87% versus 81%	Grade 3 or higher TRAEs: 75% vs. 69%	HR for disease progression or death was higher in the PD-L1-positive subgroup.	1b
Yang et al. (48) (NECTAR trial)	2024	II	locally advanced rectal cancer	50	tislelizumab and capecitabine plus long-course chemoradiotherapy	CR	CR: 40.0%	Grade 1–2 TRAEs: 52.0%; Grade 3 TRAEs:4.0%	The rates of infiltration of exhausted T cells, TAMs, PD-1-positive TAMs, PD-1-positive M2 decreased after PD-1 blockade plus CRT therapy.	2b
Ze-Rui Zhao et al. (49) (SACTION01 Trails)	2024	II	resectable non-small cell lung cancer	46	SBRT to the primary tumor followed by tislelizumab plus platinum-based chemotherapy	major pathological response	35 (76%)	Grade 3 or higher TRAEs: 26%	The treatment effect did not differ in patients with a positive or negative PD-L1 expression status.	2b

OS, overall survival; TRAEs, treatment-related adverse events; NSCLC, non-small cell lung cancer; ORR, objective response rate; DFS, disease-free survival; DCR, disease control rate; PFS, progression-free survival; CBR, clinical benefit rate; SBRT, stereotactic body radiotherapy; TACE, transarterial chemoembolization; rPFS, radiographic progression-free survival; CR, complete response. OCEBM, Oxford Centre for Evidence-Based Medicine.

Given the heterogeneity of trial designs and maturity, we classified each clinical dataset using the Oxford Centre for Evidence-Based Medicine (OCEBM) Levels of Evidence (2009) (https://www.cebm.net/) for therapeutic interventions to aid interpretation of generalizability. In brief, Level 1b denotes an individual randomized controlled trial (e.g., phase III or randomized phase II), Level 2b denotes a prospective non-randomized cohort or single-arm phase II study, and Level 4 denotes early-phase dose-escalation or exploratory case series; the assigned evidence level is shown in **Table 1**.

#### CA184-043 (ipilimumab with RT in prostate cancer treatment)

The phase III CA184–043 trial investigated the efficacy of ipilimumab, an anti-CTLA-4 antibody, in conjunction with RT for patients with metastatic castration-resistant prostate cancer (mCRPC). Although the combination did not yield an OS benefit (HR 0.85; p = 0.053) among the entire study population, subgroup analyses indicated that ipilimumab produced a consistent benefit in terms of progression-free survival and PSA responses (a confirmed ≥50% decrease in PSA levels 13% vs. 5.3% for ipilimumab vs. placebo), underscoring the critical role of patient selection (41). With extended follow-up (final database lock on July 13, 2015), Kaplan–Meier curves crossed at ~7–8 months and then separated in favor of ipilimumab, yielding a time-dependent treatment effect: piecewise HRs were 1.49 (0–5 months), 0.66 (5–12 months), and 0.66 (>12 months). Landmark OS rates at 2, 3, 4 and 5 years were higher for patients in the ipilimumab arm (25.2% vs. 16.6%; 15.3% vs. 7.9%; 10.1% vs. 3.3%; and 7.9% vs. 2.7%, respectively). The trial therefore highlights both the promise of RT–ICI strategies (possible durable benefit in a subset) and the urgent need for predictive biomarkers and rational combination approaches to improve the benefit ratio.

#### PACIFIC trial (durvalumab for patients with stage III NSCLC)

The landmark PACIFIC trial established the efficacy of consolidation treatment with durvalumab, an anti-PD-L1 agent, after concurrent chemoradiotherapy (cCRT) in patients with unresectable stage III NSCLC who had not progressed after cCRT (42). This phase III randomized trial revealed that the addition of durvalumab significantly increased both the PFS and OS compared with the placebo. Specifically, the 12- and 18-month PFS rates were 55.9% and 44.2%, respectively, for patients in the durvalumab arm versus 35.3% and 27.0%, respectively, for patients in the placebo arm. The objective response rate was higher in patients treated with durvalumab (28.4% vs. 16.0%), and the median time to death or distant metastasis was prolonged (23.2 vs. 14.6 months). Durvalumab also reduced the incidence of new metastatic lesions, including new brain metastases. The PACIFIC trial set a new standard of care for patients with unresectable stage III NSCLC and underscores the synergistic potential of RT and ICIs in improving systemic antitumor immunity.

In terms of stratification based on PD-L1 expression, a benefit was observed broadly across prespecified subgroups. Importantly, a PFS advantage with durvalumab was observed irrespective of pretreatment tumor PD-L1 expression when the prospectively tested threshold reported in the study (≥25% versus <25%) was used, although a substantial fraction of patients had an unknown PD-L1 status. Moreover, a subgroup analysis based on driver gene mutations revealed that patients with EGFR-mutated NSCLC benefitted only slightly from the PACIFIC regimen. The trial revealed key translational questions—optimal biomarker thresholds (e.g., PD-L1) and the impact of driver mutations (e.g., EGFR)—that continue to shape subsequent trials and real-world implementation.

#### CheckMate 577 (nivolumab for patients with esophageal or gastroesophageal junction cancer)

CheckMate 577 evaluated nivolumab, an anti-PD-1 agent, after chemoradiotherapy (CRT) in patients with resected (R0) stage II–III esophageal or gastroesophageal junction cancer who had residual pathological disease after neoadjuvant chemoradiotherapy and surgery. Patients were randomized 2:1 to receive nivolumab (240 mg every 2 weeks for 16 weeks, then 480 mg every 4 weeks) or a matching placebo for up to 1 year.

The trial showed a clinically and statistically significant improvement in disease-free survival of 22.4 months with nivolumab versus 11.0 months with the placebo (43). Kaplan–Meier curves demonstrated sustained separation, and the benefit was observed early and maintained over time. Nivolumab also reduced both locoregional and, importantly, distant recurrence and prolonged distant metastasis-free survival (median 28.3 vs. 17.6 months; HR 0.74). These results indicate that adjuvant anti-PD-1 therapy after multimodal local therapy substantially decreases the risk of systemic relapse in a high-risk, post-CRT population. Prespecified and *post hoc* subgroup analyses showed that the improvement in disease-free survival (DFS) was broadly consistent across histological types (adenocarcinoma and squamous cell carcinoma), nodal status, and most clinical strata. A benefit was observed irrespective of tumor cell PD-L1 expression using the assay reported in the trial (≥1% versus <1%), and *post hoc* analyses using a combined positive score (CPS) also favored nivolumab.

In summary, CheckMate 577 highlights the capacity of checkpoint inhibition to convert an otherwise surveillance-only paradigm into an active adjuvant approach that decreases distant relapse. The remaining questions important for clinical translation and tumor-immunology research include the durability of the overall survival benefit, optimal patient selection (using biomarkers such as PD-L1/CPS and molecular subgroups), the ideal timing after surgery, and how adjuvant PD-1 inhibitors should be integrated with perioperative chemotherapeutic strategies.

#### RAPPORT trial (pembrolizumab for treating oligometastatic renal cell carcinoma)

The RAPPORT trial was a prospective, multi-institutional, single-arm phase I/II study that tested the safety and efficacy of total metastatic ablation with radiotherapy followed by a short course of anti-PD-1 therapy (pembrolizumab) in patients with oligometastatic clear-cell renal cell carcinoma (ccRCC). Eligible patients had one to five metastases and ≤ two prior systemic therapies; radiotherapy was delivered to all sites (preferentially single-fraction stereotactic body radiation therapy (SBRT) 20 Gy, or 30 Gy in 10 fractions when SBRT was not feasible), and 200 mg of pembrolizumab was administered IV every 3 weeks for eight cycles (≈6 months), typically starting ~5 days after the completion of RT. Thirty evaluable patients (median age, 62 years) were treated and followed for a median of 28 months.

Efficacy indicators were encouraging for a short systemic treatment course administered in the context of complete metastasis-directed radiotherapy. The best overall responses included complete responses in 12 patients (40%) and partial responses (PRs) in 7 patients (23%), yielding an objective response rate (ORR) of 63% and a disease control rate (DCR) of 83%. Lesion-level local control was excellent: the 1- and 2-year rates of freedom from local progression (FFLP) were 94% and 92%, respectively. Systemic outcomes were promising (median PFS ≈ 15.6 months; 1- and 2-year PFS 60% and 45%; 1- and 2-year overall survival 90% and 74%, respectively), and the median duration of the response was 24 months. These results compare favorably with data for the effect of contemporaneous pembrolizumab monotherapy on more heavily pretreated or higher-burden populations reported within the discussion section of the report, although caveats about cross-study comparisons apply.

In summary, RAPPORT demonstrates that total metastatic ablation plus a limited (6-month) course of pembrolizumab is feasible, yields excellent local control and promising systemic activity, and has a tolerable safety profile in a highly selected oligometastatic ccRCC population. Key limitations include the single-arm design, small sample size, and selection bias toward patients with a favorable/intermediate-risk, low-volume disease; consequently, randomized evaluation and biomarker studies are needed to define which patients derive a durable systemic benefit, whether multisite ablation is superior to single-site RT for inducing systemic immunity, and how this approach should be integrated with modern first-line combination regimens (44).

#### CheckPAC trial (nivolumab with or without ipilimumab in combination with SBRT for patients with refractory mPC)

CheckPAC was a prospective, randomized phase II study that evaluated the addition of SBRT (single-fraction 15 Gy to one measurable lesion) to nivolumab with or without ipilimumab to treat patients with refractory metastatic pancreatic ductal adenocarcinoma (mPC). Patients were randomized 1:1 to receive SBRT + nivolumab (arm A) or SBRT + nivolumab/ipilimumab (arm B); treatment continued for up to 52 weeks or until progression/toxicity occurred. The trial used Simon’s two-stage design independently for each arm with the clinical benefit rate (CBR; CR/PR/SD by RECIST v1.1) as the primary endpoint and enrolled 84 treated patients (41 in arm A and 43 in arm B).

Efficacy indicators favored the combination that included CTLA-4 blockade. The prespecified CBR threshold was met in arm B: the CBR was 37.2% (95% CI, 24.0–52.1) with SBRT/nivolumab/ipilimumab versus 17.1% (95% CI, 8.0–30.6) with SBRT/nivolumab. The objective response rates were 14.0% in arm B and 2.4% in arm A. Despite the higher CBR/ORR in the dual-checkpoint cohort, the time-to-event endpoints were short in this heavily pretreated population: the median progression-free survival was 1.6 months (arm B) versus 1.7 months (arm A), and the median overall survival was 3.8 months for patients in both arms (45).

CheckPAC demonstrated that adding single-fraction SBRT to dual checkpoint blockade can produce clinically meaningful responses in a disease that has been historically refractory to immunotherapy and that the addition of ipilimumab increased the proportion of patients who achieved clinical benefit. However, the trial was not powered for a formal between-arm comparison; SBRT was administered to patients in both arms (and thus the independent contribution of RT cannot be isolated), follow-up was limited in this highly selected, rapidly progressive population, and biomarkers remained exploratory. These caveats underscore the need for randomized, larger trials that (1) include control arms without RT (2), explore optimal dose/fractionation and multisite versus single-site irradiation, and (3) prospectively validate translational biomarkers such as cytokine dynamics to guide patient selection and sequencing.

#### DOLPHIN trial (durvalumab plus concurrent RT for NSCLC treatment)

The DOLPHIN trial investigated the efficacy of durvalumab combined with concurrent RT in patients with PD-L1-positive, unresectable, or locally advanced NSCLC or postoperative locoregional recurrence. Key eligibility criteria included an ECOG score of 0–1 and a PD-L1 TPS ≥ 1% (SP263, central review). Treatment comprised involved-field RT of 60 Gy in 30 fractions (3D-CRT or IMRT; elective nodal irradiation was omitted) initiated on day 1 of treatment with 10 mg/kg durvalumab every 2 weeks, with durvalumab continued for up to 12 months. The primary end point was the 12-month PFS rate according to an independent central review (ICR); the secondary end points included PFS, the ORR, treatment completion, and safety with prospective RT quality assurance and DSMC oversight. The sample size (n = 35) was based on a 12-month PFS null value of 28% and an alternative value of 50%.

The trial produced promising results, with a 12-month PFS rate of 72.1% (95% CI, 59.1%–85.1%) and a median PFS of 25.6 months. The trial also reported a confirmed ORR of 90.9% (95% CI, 75.7%–98.1%), highlighting the potential of combining durvalumab with RT to treat NSCLC. Despite a promising 12-month PFS rate of 72.1%, grade ≥3 pneumonitis occurred in 11.8% of patients, highlighting the trade-off between efficacy and toxicity. Compared with the PACIFIC trial, the DOLPHIN trial did not require chemotherapy but achieved comparable PFS rates, suggesting that chemo-sparing regimens may be feasible in selected populations. However, the absence of a control arm limits definitive conclusions (46).

#### ENGOT-cx11/GOG-3047/KEYNOTE-A18 trial (pembrolizumab for patients with high-risk, locally advanced cervical cancer)

The ENGOT-cx11/GOG-3047/KEYNOTE-A18 trial is a global, randomized, double-blind, placebo-controlled phase III study evaluating the efficacy of the addition of pembrolizumab to definitive CRT in newly diagnosed, high-risk patients with locally advanced cervical cancer. Eligible patients (≥18 years) from 176 centers across 30 countries were randomized 1:1 to receive pembrolizumab or the placebo concurrently with CRT (five cycles of 200 mg of pembrolizumab or the placebo every 3 weeks plus CRT), followed by 15 maintenance cycles of 400 mg of pembrolizumab or the placebo every 6 weeks. Stratification was performed by radiotherapy.

At the data cutoff (January 9, 2023), the median follow-up was 17.9 months (IQR 11.3–22.3) in both treatment groups, the pembrolizumab arm achieved a statistically significant improvement in PFS, with a 24-month PFS rate of 68% versus 57% in the placebo group (HR, 0.70; 95% CI, 0.55–0.89; p=0.0020). The overall survival rate at 24 months was 87% in the pembrolizumab–CRT group and 81% in the placebo–CRT group, although this interim OS analysis did not meet the threshold for statistical significance. The hazard ratio (HR) for death was 0.73 (95% CI 0.49–1.07); these data did not meet the threshold for statistical significance. The rates of grade 3 or higher adverse event rates were 75% in the pembrolizumab–CRT group and 69% in the placebo–CRT group (47).

In summary, KEYNOTE-A18 demonstrated that pembrolizumab combined with CRT significantly prolongs PFS in newly diagnosed, high-risk patients with locally advanced cervical cancer, establishing a new benchmark for first-line treatment in this setting. This benefit was observed across a large, international cohort, supporting its generalizability. While the OS data are not complete, the trend toward improved survival supports the use of pembrolizumab–CRT as a new standard-of-care treatment. Ongoing priorities include extended OS follow-up, biomarker-driven analyses (e.g., PD-L1 expression and HPV subtype), and strategies for optimizing integration with systemic or consolidative therapies to further improve the outcomes.

#### NECTAR trial (tislelizumab for patients with locally advanced rectal cancer)

The NECTAR trial was a prospective, multicenter, single-arm phase II study designed to evaluate the efficacy and safety of PD-1 blockade in combination with long-course CRT for patients with microsatellite stable (MSS) or mismatch repair-proficient (pMMR) locally advanced rectal cancer (LARC). From June 2021 to November 2022, 50 eligible patients (cT3–4aN0M0 and cT1–4aN1–2M0) were enrolled at six centers in China. Treatment consisted of long-term radiotherapy with concurrent capecitabine and tislelizumab, followed by total mesorectal excision 6–12 weeks after the completion of CRT.

The trial met its primary endpoint; the pathological complete response (pCR) rate was 40.0% (20/50; 95% CI, 27.6–53.8), which compares favorably with historical rates of 10–20% for neoadjuvant CRT alone. Additionally, 30.0%, 18.0%, and 4.0% of patients achieved AJCC tumor regression grades of 1, 2, and 3, respectively. Among the patients who underwent surgery, the R0 resection rate was 100%, with sphincter preservation achieved in 89.1% of patients. The objective response rate based on RECIST v1.1 was 76.1%, including 39.1% with complete responses and no progressive disease. Early translational analyses revealed reduced numbers of exhausted T cells, tumor-associated macrophages, and PD-1-positive M2 macrophages following treatment, suggesting that tislelizumab plus CRT remodeled the immunosuppressive tumor microenvironment in MSS/pMMR tumors (48).

In summary, the NECTAR trial demonstrated that tislelizumab combined with long-course CRT is a feasible and effective neoadjuvant regimen for MSS/pMMR LARC, with a substantially higher pCR rate than conventional CRT. These findings highlight the potential for PD-1 blockade to convert immunologically “cold” MSS/pMMR rectal tumors into “hot” responsive tumors. However, the single-arm design and limited sample size limit the generalizability of the results. Ongoing phase III randomized trials (e.g., NCT05245474) are expected to validate these findings, optimize patient selection strategies, and determine whether long-term survival outcomes and organ preservation rates are significantly improved by this approach.

#### Other notable trials

SACTION01 (resectable NSCLC) was a single-center, single-arm phase II trial testing whether preoperative SBRT could immunomodulate tumors and increase the efficacy of neoadjuvant immunochemotherapy in patients with resectable stage IIA–IIIB NSCLC. Patients received SBRT to the primary tumor (24 Gy in three fractions) followed by two 21-day cycles of tislelizumab (200 mg) plus platinum-based chemotherapy (carboplatin–pemetrexed or nab-paclitaxel), with resection planned 4–6 weeks later. The primary endpoint was a major pathological response (MPR; ≤10% residual viable tumor). Among the 46 intention-to-treat patients, MPR was achieved in 35 (76%, 95% CI 61–87), which considerably higher than historical rates with immunochemotherapy alone. Grade ≥3 treatment-related adverse events occurred in 26% (most commonly neutropenia), and one treatment-related death due to neutropenia was noted; no deaths occurred at the 90-day post-operative time point. These data suggest that subablative SBRT can be a feasible immune primer that enhances the pathological response before surgery, warranting confirmation in a randomized trial (49).

ICE-PAC (metastatic castration-resistant prostate cancer) was a multicenter, single-arm phase II study evaluating PD-L1 blockade with focal high-dose radiotherapy in heavily pretreated patients with mCRPC. Thirty-one evaluable men received 10 mg/kg avelumab every 2 weeks for 24 weeks; SBRT (20 Gy in a single fraction) was delivered to one or two lesions within 5 days before the first and second avelumab doses. The primary endpoint—the disease control rate (DCR: confirmed CR/PR of any duration or SD/non-PD ≥6 months)—was 48% (15/31; 95% CI 30–67). In patients with measurable disease, the ORR was 31% (5/16), with a comparable 33% ORR in nonirradiated lesions, indicating out-of-field activity. The median radiographic PFS was 8.4 months (95% CI 4.5–NR), and the median OS was 14.1 months (95% CI 8.9–NR). Grade 3–4 treatment-related adverse events occurred in 16% of the patients; three patients (10%) required high-dose corticosteroids. Exploratory plasma analyses suggested that changes in androgen receptor (AR) levels were associated with a lower DCR (22% vs. 71%), underscoring the genomic determinants of ICI resistance in prostate cancer. Limitations include the small sample size and the absence of a control arm (50).

START-FIT (locally advanced, unresectable hepatocellular carcinoma) was a single-arm, phase II conversion therapy trial that integrated sequential locoregional therapy with immunotherapy. Patients underwent conventional transarterial chemoembolization (TACE; day 1), followed by SBRT to all liver lesions (27.5–40.0 Gy in five fractions; day ~28) and then avelumab (10 mg/kg) every 2 weeks starting ~14 days after SBRT. The primary endpoint—amenability to curative treatment—was met in 18/33 (55%) patients: four patients (12%) underwent resection or radiofrequency ablation, and an additional 14 (42%) achieved a sustained radiological complete response and elected surveillance. Grade ≥3 treatment-related adverse events occurred in 33% of patients overall, driven mainly by transient post-TACE transaminitis (15%), and grade ≥3 immune-related events occurred in 15% of patients (hepatitis and dermatitis). These findings support RT–ICI–TACE triplet therapy as a bridge to curative strategies in patients with liver-limited disease, meriting randomized validation (51).

Taken together, these trials indicate both the breadth and complexity of RT–ICI integration across diverse tumor types and treatment settings. From large, practice-changing phase III studies such as PACIFIC, CheckMate 577, and KEYNOTE-A18 to innovative phase II designs including CheckPAC, DOLPHIN, NECTAR, and other notable exploratory efforts, the data collectively highlight that radiotherapy can potentiate the effects of immune checkpoint blockade by increasing systemic antitumor immunity, enhancing pathological response, and, in selected cases, converting previously unresectable disease to curative opportunities. Moreover, these studies underscore key challenges that remain unresolved: heterogeneity in efficacy across tumor histologies and molecular subtypes, the balance between enhanced efficacy and immune-related toxicity, and the lack of validated biomarkers to guide patient selection and sequencing. Future research should prioritize randomized, biomarker-driven trials, investigate optimal RT doses and fractionation schedules, and explore rational multimodal combinations to fully harness the curative potential of RT–ICI strategies.

## Optimizing radiation therapy for immunotherapy

**Figure 2:**
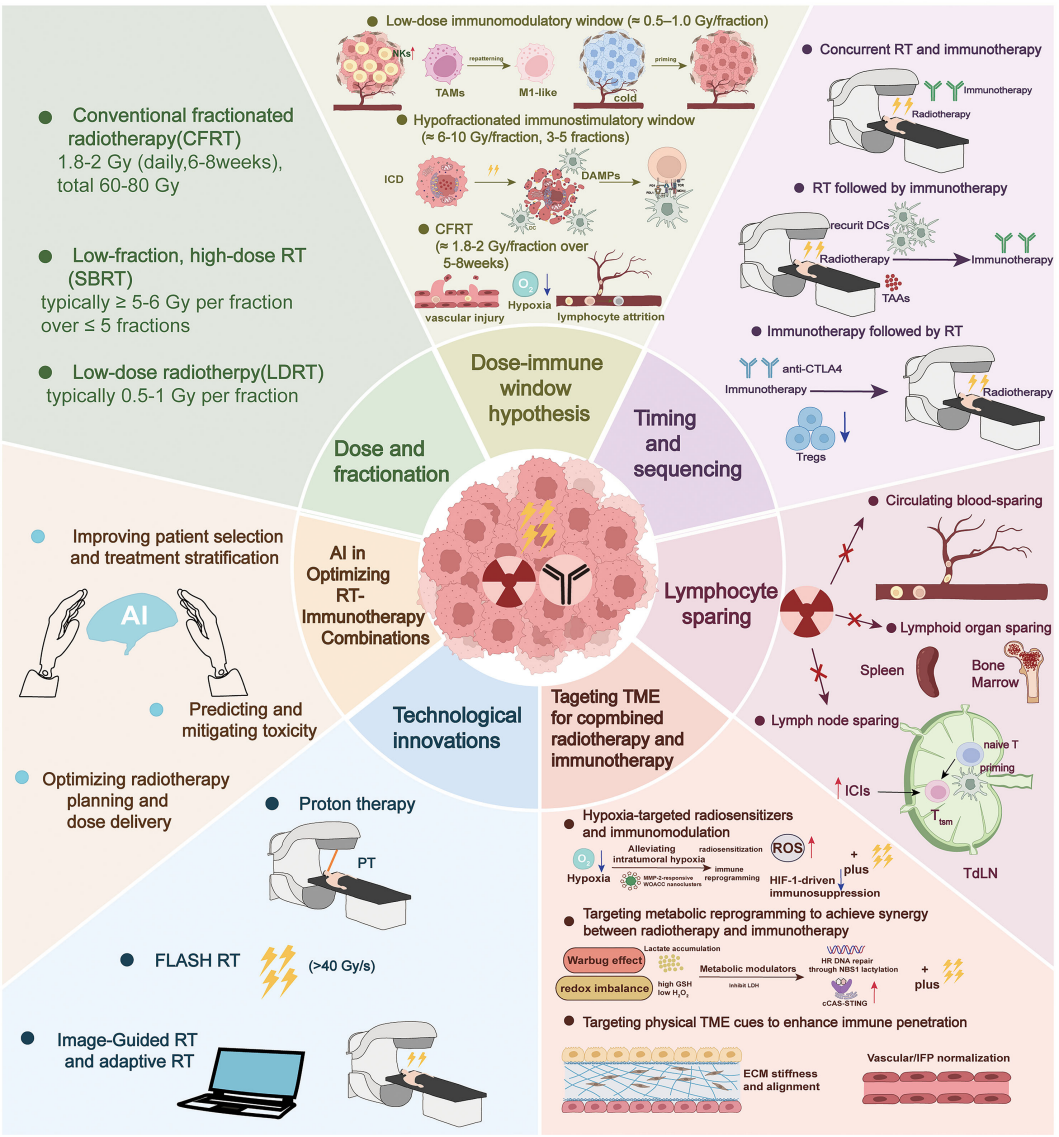
Framework for optimizing radiotherapy (RT) to synergize with immunotherapy. Schematic overview integrating six strategy domains that together maximize RT–immunotherapy synergy while minimizing immune suppression. Dose and fractionation: concept of “dose–immune windows,” contrasting CFRT (≈1.8–2 Gy/fraction over 6–8 weeks) with SBRT (≥5–6 Gy/fraction in ≤5 fractions) and LDRT (≈0.5–1 Gy/fraction), highlighting their distinct effects on ICD/DAMP release, vascular injury, hypoxia, and lymphocyte attrition. Timing and sequencing: options for concurrent delivery, RT→immunotherapy, or immunotherapy→RT, chosen to align with waves of antigen release, PD-L1 induction, and dendritic-cell licensing. Lymphocyte sparing: principles to limit irradiation of circulating blood and lymphoid reservoirs (bone marrow, spleen) and to preserve tumor-draining lymph nodes (TDLNs) that seed systemic recall responses. Targeting the TME: radiosensitization and immune reconditioning via hypoxia modulation, metabolic reprogramming (e.g., lactate/GSH/ROS balance), and normalization of physical barriers (ECM stiffness/alignment and vascular/IFP). Technological innovations: proton therapy to reduce integral dose, FLASH-RT to spare normal tissues while favorably polarizing myeloid cells, and image-guided/adaptive RT to tighten margins and curtail the low-dose bath. AI enablement: data-driven tools for patient selection/stratification, toxicity prediction, and planning/delivery optimization. Lightning bolts denote RT; upward red arrows indicate up-regulation; icons depict the listed tissues, cells, and devices.

The integration of RT with immunotherapy has emerged as a transformative approach in oncology, leveraging the ability of RT to modulate the TME and immune responses to enhance systemic antitumor immunity. However, the success of this combination strongly depends on optimizing RT parameters to maximize synergistic interactions while minimizing immunosuppressive effects. This section focuses on key strategies to refine RT for improved compatibility with immunotherapy, exploring critical determinants such as the dose and fractionation schedules, the timing and sequencing of treatments, the preservation of lymphoid structures, targeted modulation of the TME, and cutting-edge technological and AI-driven innovations (**Figure 2**). By dissecting the mechanistic interplay between RT and immune function and synthesizing preclinical and clinical evidence, this section aims to provide a framework for tailoring RT to unlock the full potential of immunotherapeutic agents in diverse cancer contexts.

### Dose and fractionation

The choices of dose and fractionation emerge as among the most important factors influencing the interplay between RT and the immune system. The interplay between the radiation dose/fractionation and antitumor immunity is characterized by complex, dose-dependent effects on the TME, immune cell function, and systemic immune responses (52). In this section, the mechanisms underpinning the effects of high-dose, low-dose, and conventional fractionated radiotherapy are integrated with clinical evidence, highlighting synergistic and antagonistic effects in combination with immunotherapy.

#### Conventional fractionated radiotherapy: immunosuppression and limited synergy

CFRT is a standard radiation modality involving the administration of daily doses of 1.8–2 Gy per fraction over 6–8 weeks, with total doses of 60–80 Gy, and it is typically used for treating multiple solid tumors, such as locally advanced prostate cancer and non-small cell lung cancer. In practice, ICI is often initiated 3–6 weeks after the completion of CFRT, as exemplified by the PACIFIC paradigm (52, 53). However, Compared with SBRT, CFRT relies mainly on gradual reoxygenation during prolonged treatment courses, which may be insufficient to overcome tumor hypoxia (54). This process triggers multiple immunosuppressive effects, such as impaired trafficking of cytotoxic CD8^+^ T cells and NK cells due to vascular damage (55, 56); the polarization of tumor-associated macrophages toward M2-like phenotypes that secrete anti-inflammatory cytokines (57); the promotion of T-cell exhaustion and conversion to Treg-like cells (58); and the activation of autophagy/stress pathways, increasing cancer cell resistance to immune-mediated cytotoxicity (59).

Unfortunately, conventional fractionation has been shown to reduce the number of tumor-infiltrating CD8^+^ CTLs during treatment, likely due to the radiation-induced death of these effector immune cells. In preclinical models, extending a hypofractionated single dose to a daily fractionated schedule markedly attenuated intratumoral CD8^+^ T-cell infiltration (from ~70% after a single 30 Gy dose to ~4–8% with 3 Gy × 10), which coincided with inferior tumor control and expansion of myeloid suppressor populations. These data support the hypothesis that prolonged fractionation may compromise antitumor T-cell function, at least in some settings (60).

In addition to local TIL dynamics, CFRT exerts systemic lymphotoxic effects through repeated irradiation of the circulating lymphocyte pool. Biophysical modeling of a standard glioblastoma plan (60 Gy in 30 fractions to an 8-cm target) estimated that by the end of treatment, ~99% of circulating lymphocytes had received ≥0.5 Gy, with a mean dose to circulating cells (DCC) of ~2.2 Gy. Notably, the DCC was largely insensitive to the delivery technique (IMRT vs. 3D-CRT) or dose rate but scaled strongly with the planned target volume. Lymphocytes are among the most radiosensitive normal cells (D10 ≈3 Gy), with CD4^+^ and CD8^+^ subsets showing comparable radiosensitivity *in vitro* (e.g., D10 ~3.3–3.8 Gy). Collectively, these observations suggest that cumulative, fraction-by-fraction exposure of circulating lymphocytes is a principal driver of radiation-induced lymphopenia (RIL) (61–63). Clinical evidence underscores the relevance of these mechanisms. In patients with locally advanced pancreatic cancer, conventional chemoradiation (median of 50.4 Gy in 1.8 Gy fractions) resulted in severe lymphopenia (TLC < 500 cells/mm³) in 71.7% of patients at 1 month, compared with 13.8% after SBRT (33 Gy in 5 fractions); 46.0% of CRT patients remained severely lymphopenic at 2 months. Importantly, a higher posttreatment TLC was independently associated with prolonged survival, and the disparity in lymphopenia between SBRT and CRT persisted after accounting for chemotherapy exposure, which is consistent with modeling predictions that the fraction number and field size govern the RIL risk (64).

In addition to PTV-driven blood irradiation, inadvertent exposure of secondary lymphoid organs—particularly the spleen—contributes to lymphopenia. In gastric cancer CRT, a higher mean splenic dose (MSD) and larger low-to-intermediate dose volumes (e.g., V20/V30/V40) were associated with grade ≥ 3 lymphopenia, and an MSD > 40 Gy increased the odds of severe lymphopenia. These findings, together with similar observations in upper abdominal malignancies, support treating the spleen as an organ at risk and motivate spleen-sparing planning when feasible (65).

Therefore, when CFRT is unavoidable, fewer fractions, smaller PTVs, and spleen sparing should be prioritized, and the parameters being managed (serial TLC + spleen DVH) should be measured to mitigate RIL while preserving compatibility with immunotherapy.

#### Low-fraction, high-dose RT: potent immunogenicity via ICD and TME remodeling, with superior clinical outcomes

SBRT delivers high biological doses in few fractions (typically ≥5–6 Gy per fraction over ≤5 fractions) and, compared with conventional fractionation, more potently elicits ICD along with the exposure/release of calreticulin, ATP, and HMGB1, thereby increasing the DC uptake and cross-presentation of tumor antigens. These ICD hallmarks are dose dependent, providing a mechanistic bridge from localized irradiation to systemic antitumor priming (66–68).

Beyond ICD, SBRT promotes antigen cross-presentation in draining lymph nodes and increases antigen-experienced/effector memory CD8^+^ T-cell pools, offering a mechanistic rationale for the synergistic effects with PD-1 blockade (69). In parallel, SBRT reshapes the TME toward T-cell recruitment by upregulating CXCL9/10/16 and type-I interferon-related programs, with an increased TCR repertoire diversity and PD-L1 expression observed in paired human lung tumor samples shortly after SBRT; notably, CD8^+^ infiltration may lag in the first week, emphasizing the importance of the sampling window when on-treatment biopsies are being interpreted (70). Systemically, prospective immune monitoring in patients with prostate cancer indicates that 3-fraction SBRT (e.g., 40 Gy/3f) increases central and effector memory CD8^+^ T cells and decreases Treg frequencies, improving the CD8/Treg balance compared with conventionally fractionated RT (71). Concordantly, the expansion of circulating CX3CR1^+^ CD8^+^ T cells has emerged as a dynamic blood-based biomarker of effective ICI responses, which aligns with RT-driven trafficking cues (72). Clinically, in the randomized PEMBRO-RT trial, prior SBRT doubled the 12-week ORR to pembrolizumab (36% vs 18%), with PFS/OS gains most evident in PD-L1–negative tumors; SBRT was well tolerated (73). Consistent multi-omic correlative data further show that patients with immunologically cold tumors experience longer PFS with SBRT-primed pembrolizumab, reinforcing the biological and clinical rationale for favoring SBRT over CFRT when integrating checkpoint blockade (74).

However, when combining SBRT with immune checkpoint blockade, per-fraction doses should be constrained: a central upstream signal connecting SBRT-induced damage to antitumor immunity is mislocalized cytosolic dsDNA, which serves as a self-derived danger signal sensed by cGAS, producing cGAMP to activate STING and drive type-I interferon programs that license Batf3-dependent type-1 conventional dendritic cells (cDC1s) and prime CD8^+^ T-cell responses. Crucially, this dsDNA signal is dose- and fractionation-dependent: in multiple tumor models, single fractions greater than ~12–18 Gy induce the expression of the exonuclease TREX1, which degrades radiation-induced cytosolic DNA, blunting cGAS–STING activation and DC licensing; in contrast, repeated subthreshold fractions (e.g., ~8 Gy × 3) sustain pulsatile cytosolic DNA and amplify IFN-β/ISG programs, restoring systemic tumor rejection when combined with checkpoint blockade. The loss of cGAS/STING or TREX1 overexpression abrogates these abscopal effects, underscoring that the fractionation benefit requires intact DNA sensing. Regarding the design implications, when systemic immune activation is a goal, hypofractionated schedules that are lower than strong TREX1 induction thresholds and still maintain local control are preferred, as the precise window is tumor- and context-dependent (75, 76).

Therefore, SBRT leverages ICD, cGAS–STING signaling, and chemokine remodeling to prime systemic immunity; fractionation within the TREX1-sparing window (e.g., ~8 Gy×3) appears optimal for combination with ICIs, and judicious LDRT to nonindex lesions may further unlock abscopal control, but attention to the timing of sampling and disease context remains essential.

#### Low-dose radiotherapy (LDRT, <2 Gy per fraction): subtle TME reprogramming through sustained immunomodulation, with the potential for synergistic effects with combination therapies

LDRT (typically 0.5–1 Gy per fraction) reprograms the TME through sustained immunomodulation with minimal direct cytotoxicity, thereby prioritizing immune activation over tumor cell death. In murine models, single 1-Gy exposure triggered acute cellular stress (calreticulin exposure, γH2AX foci) without meaningful tumor growth inhibition but broadly activated the type I/II interferon signaling, complement, and IL-6/JAK/STAT3 pathways. These changes coincide with the upregulation of T-cell-recruiting chemokines (e.g., CXCL9/CXCL10) and an influx of lymphocytes into the intraepithelial tumor compartment (77–79).Myeloid and dendritic compartments are similarly remodeled. Preclinical LDRT (≈1 Gy × 1–2) increases the number of M1-polarized TAMs, increases NK-cell infiltration, and reduces TGF-β levels, collectively fostering a proinflammatory milieu; these effects likely involve innate pattern recognition signaling (including TLR pathways) (78).

A distinct line of evidence supports intestinal LDRT (ILDR) as an immunological adjuvant to PD-L1 blockade. Delivering ~1 Gy to segments of the small bowel (without direct tumor targeting) promotes the egress of CCR7^+^PD-L1^+^ regulatory DCs (mregDCs) from mesenteric lymph nodes to tumor-draining lymph nodes, increasing CD8^+^ T-cell activation. This effect is associated with the presence of *Christensenella* spp. (including an ~30% fecal prevalence of *C. minuta* in treated patients) and metabolic reprogramming that decreases lactate levels while increasing the levels of secondary bile acids (e.g., deoxycholic/ursodeoxycholic acid) and indole derivatives that enhance DC antigen presentation (80).

Clinically, in the phase I RACIN study (NCT03728179), patients with “immune-desert” tumors (intraepithelial CD8^+^ < 5 cells/HPF) received weekly LDRT (0.5–1 Gy/fraction to select lymphoid/abdominopelvic volumes) plus nivolumab/ipilimumab with low-dose cyclophosphamide and aspirin. According to iRECIST, the ORR was 12.5% (1/8 PR), and the DCR was 87.5% (7/8 PR or SD); importantly, progression occurred predominantly at nonirradiated sites, whereas several irradiated lesions shrank, highlighting the field dependence. In addition to single-arm trials, dosimetric analyses across SBRT-ICI datasets suggest that specific abdominopelvic “volume windows” for incidental 0.25–4 Gy exposure (e.g., small intestine ~7–588 cc; colon ~1,031–5,766 cc) are associated with improved survival (e.g., higher OS24), and the ILDR emerged as an independent prognostic factor (HR ~0.22), supporting the concept that controlled low-dose exposure of immunologically active viscera can systemically potentiate ICI (78, 80).

Collectively, LDRT, particularly when coordinated with checkpoint blockade, can convert immune-cold phenotypes through chemokine induction, DC/cDC1-dependent cross-priming, and macrophage/NK repatterning, with the gut-targeted ILDR activating an additional microbiota–metabolite axis to amplify responses. Prospective studies should refine the dose and fractionation (≈0.5–1 Gy), select immunologically relevant organs/volumes, and rationalize multilesion coverage to limit out-of-field escape while preserving safety (78, 81).

### Dose–immune window hypothesis

**Figure 3:**
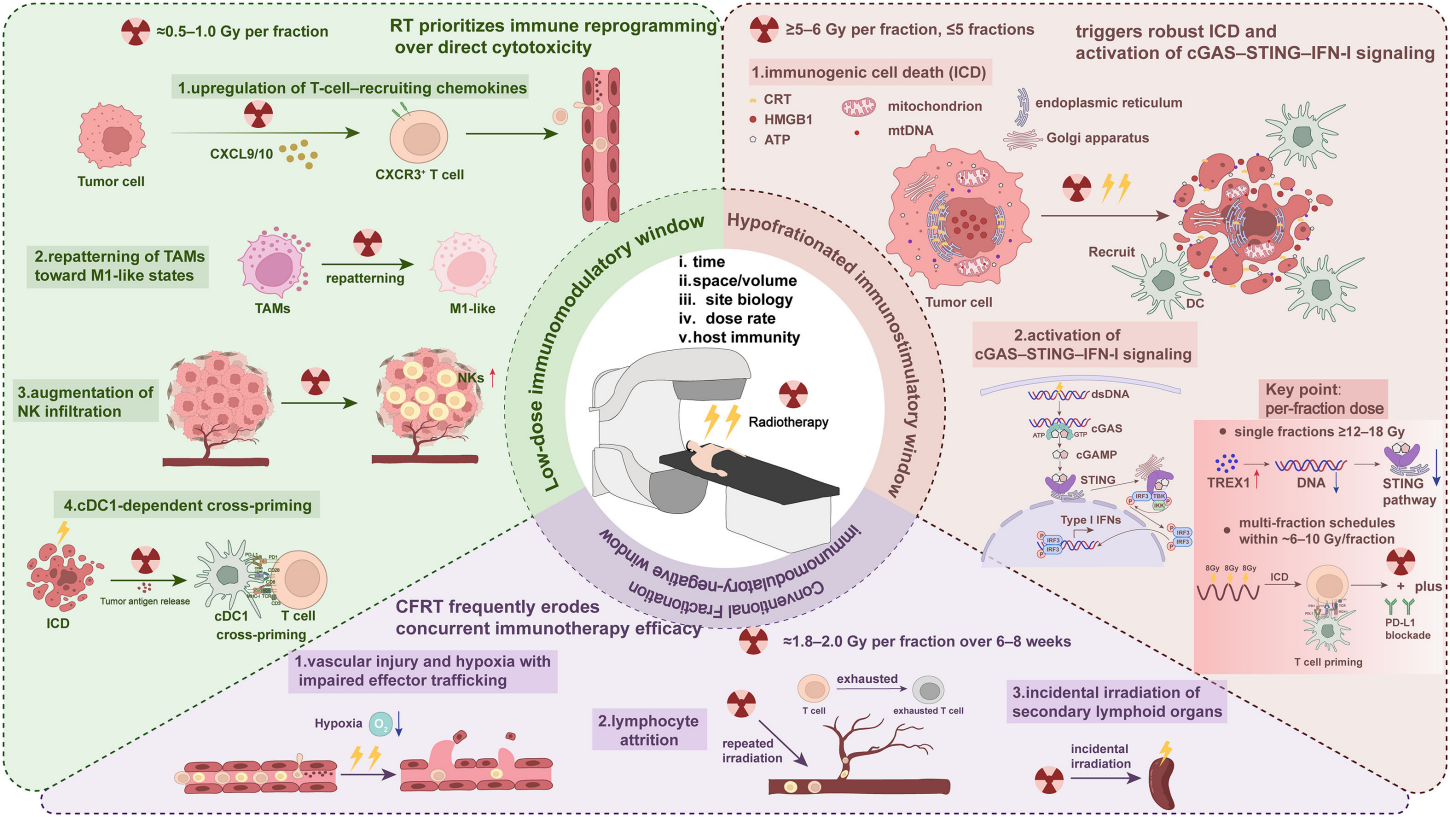
The dose–immune window hypothesis for integrating radiotherapy (RT) with immunotherapy. Conceptual schema depicting two operational synergy windows and a fractionation region that is generally unfavorable for concurrent immunotherapy. Window I—Low-dose immunomodulatory window (≈0.5–1.0 Gy per fraction): Sub-Gray RT prioritizes immune reprogramming over direct cytotoxicity, characterized by (1) up-regulation of T-cell–recruiting chemokines (e.g., CXCL9/10) and enhanced endothelial adhesion (2); repatterning of tumor-associated macrophages toward M1-like states (3); increased NK-cell infiltration; and (4) cDC1-dependent cross-priming after immunogenic stress. Window II—Hypofractionated immunostimulatory window (≥5–6 Gy per fraction, ≤5 fractions): Hypofractionation triggers robust immunogenic cell death (CRT/ATP/HMGB1 release), augments cross-presentation in draining lymph nodes, and activates cGAS–STING–IFN-I signaling from radiation-induced cytosolic DNA/micronuclei. A key constraint is TREX1 induction: single fractions ≥12–18 Gy up-regulate TREX1 and degrade cytosolic DNA, attenuating STING activation; multi-fraction schedules within ~6–10 Gy/fraction (e.g., 8 Gy × 3) preserve pulsatile DNA sensing and align with PD-(L)1 blockade. Window III—Conventional Fractionation immunomodulatory-negative window (≈1.8–2.0 Gy per fraction over 6–8 weeks): Protracted CFRT often erodes concurrent ICI efficacy via (1) vascular injury/hypoxia with impaired effector trafficking (2), lymphocyte attrition from repeated irradiation of the circulating pool (dose to circulating cells), and (3) incidental irradiation of secondary lymphoid organs (e.g., spleen). Lightning bolts denote RT.

Building on the mechanistic and clinical evidence summarized in Dose and fractionation, we formalize a dose–immune window framework that explains why RT can either potentiate or undermine the effects of immunotherapy, depending on the per-fraction dose, number of fractions, target volume, dose rate, anatomic site, and sequencing. Rather than a single optimum, RT–immune interactions exhibit nonlinear, windowed behavior, with two operational synergy windows and a fractionation region that is generally unfavorable for concurrent immunotherapy (**Figure 3**).

Window I—Low-dose immunomodulatory window (≈0.5–1.0 Gy per fraction): At sub-Gray doses, RT prioritizes immune reprogramming over direct cytotoxicity. Hallmark effects include the upregulation of T-cell-recruiting chemokines (e.g., CXCL9/10), repatterning of TAMs toward M1-like states, increase in NK infiltration, and cDC1-dependent cross-priming that can convert immune-cold phenotypes. Strategically exposing immunologically active viscera (e.g., intestinal segments; intestinal low-dose radiotherapy, ILDR) can additionally leverage gut microbiota–metabolite axes to amplify antigen presentation in tumor-draining nodes. Operationally, 0.5–1.0 Gy/fraction is the most consistent reprogramming zone; 1–2 Gy/fraction behaves as a gray area in which immune effects become more contingent on the volume, organ, and cumulative dose.

Window II—Hypofractionated immunostimulatory window (≥5–6 Gy per fraction over ≤5 fractions): Hypofractionation drives ICD with the release of DAMPs (calreticulin, ATP, and HMGB1), promotes antigen cross-presentation in draining lymph nodes, and activates cGAS–STING–IFN-I signaling via cytosolic DNA generated by DNA damage and micronuclei. Crucially, the per-fraction dose determines whether the cytosolic DNA signal persists: single fractions ≥12–18 Gy induce TREX1 expression, inducing cytosolic DNA degradation and attenuating STING activation. Thus, multifraction schedules with ~6–10 Gy/fraction (e.g., 8 Gy × 3) preserve pulsatile DNA sensing and better align with concurrent PD-L1 blockade when systemic T-cell priming is desired.

Window III—Conventional Fractionation immunomodulatory-negative window (≈1.8–2.0 Gy per fraction over 6–8 weeks): Protracted CFRT frequently reduces the efficacy of concurrent immunotherapy by combining (i) vascular injury and hypoxia with impaired effector trafficking; (ii) lymphocyte attrition from repeated irradiation of the circulating pool, as captured by the DCC; and (iii) the incidental irradiation of secondary lymphoid organs (e.g., a higher mean spleen dose). Importantly, this region is not a universal “null”: sequential ICI after CFRT/CRT can still be effective (e.g., consolidation paradigms). The “unfavorable” label applies specifically to concurrent combinations unless lymphocyte-sparing and organ-at-risk (OAR)-sparing strategies substantially mitigate these liabilities.

Modulators of the windows: The position and width of each window are shifted by (i) the time (interfraction interval, on-treatment vs. post-RT ICI start), (ii) space/volume (PTV size, low-dose bath, coverage of nonindex lesions with LDRT), (iii) site biology (liver and brain vs. lung/lymph-node milieus), (iv) dose rate (with ultrahigh dose-rate/FLASH effects under investigation), and (v) host immunity (baseline ALC/NLR, TIL density, microbiome and metabolomic context). These factors should be explicitly integrated into planning and trial design.

### Timing and sequence

The timing and sequence of RT relative to immunotherapy are critical determinants of efficacy. Aligning RT with dynamic waves of tumor antigen release, PD-L1 induction, cytosolic DNA sensing, and dendritic cell licensing can maximize synergy while minimizing counterregulatory pathways (40).

#### Concurrent RT and immunotherapy

Concurrent delivery of RT with immune checkpoint inhibitors can harness RT-triggered immune remodeling while pre-empting adaptive resistance. Mechanistically, ionizing radiation induces antigen release and type I interferon signaling via cGAS–STING signaling and related DNA damage responses but also upregulates the expression of inhibitory ligands such as PD-L1, which can blunt cytotoxic T-cell function. Pairing RT with anti-PD-L1 therefore acts less to “prevent exhaustion” than to counteract RT-induced adaptive immune resistance and sustain effector activity (82).

Preclinical evidence supports true concurrency. In bladder cancer models, irradiation increased PD-L1 expression both *in vitro* and *in vivo*, with PD-L1 expression peaking ~72 hours after RT and decreasing by day 7; initiating anti-PD-L1 therapy around the RT window prolonged the delay in tumor growth and increased tumor cell death, which is consistent with RT-driven antigenicity but PD-L1-mediated counterregulation (83). Clinical data illustrate the feasibility of this approach. In the single-arm phase II DOLPHIN trial (PD-L1-positive, unresectable stage III NSCLC), concurrent curative-intent RT (60 Gy) plus durvalumab followed by durvalumab maintenance therapy yielded a 12-month PFS of 72.1% and a confirmed ORR of 90.9% by independent review, with rapid and deep responses suggesting immediate immune activation. Nonetheless, the interpretation is tempered by the nonrandomized design and selected population (46).

Toxicity requires proactive mitigation. With respect to the thoracic field, pneumonitis/radiation pneumonitis was frequent (any-grade 67.6%; grade 3–4 11.8%), typically peaking 8–12 weeks after treatment initiation and underscoring the need for careful lung and heart dosimetry, vigilant symptom surveillance, and early management algorithms when concurrent schedules are adopted (46, 84). When CTLA-4 blockade is employed, the risk of immune-mediated colitis warrants stringent monitoring under concurrent schedules, although contemporary series suggest overall feasibility in selected settings (85).

#### RT followed by immunotherapy

Delivering immunotherapy after RT capitalizes on a transient, RT-induced window of immune priming characterized by the release of tumor-associated antigens (TAAs), upregulation of MHC class I on tumor cells, and enhanced cross-presentation by DCs within ~48 hours, which subsequently wanes over the following days (86). This biology provides a mechanistic rationale for administering costimulatory agonists and checkpoint inhibitors after RT rather than before it.

For costimulatory agonists such as anti-OX40, administration shortly after RT—approximately 1 day—appears to be optimal, coinciding with a surge in antigen presentation and the transient upregulation of OX40 expression on activated T cells. In multiple murine models, administering anti-OX40 antibodies after RT increased CD4^+^ and CD8^+^ T-cell expansion and produced robust abscopal control, whereas induction or concurrent schedules were inferior. Mechanistically, RT increased OX40 expression on intratumoral/splenic CD4^+^ T cells and expanded CD103^+^ cross-presenting DCs within ~48 hours, aligning the peak activity of the agonist with the post-RT priming phase (87–89).

With respect to PD-1/PD-L1 blockade, preclinical data consistently favor post-RT administration. In syngeneic models, anti-PD-1 therapy delivered after local RT expanded polyfunctional intratumoral CD8^+^ T cells and reprogrammed the PD-1^hi^CD38^lo^Tcf1^hi^ subset, resulting in potent abscopal responses (89). Conversely, pre-RT anti-PD-1 therapy sensitized CD8^+^ T cells to radiation-induced DNA damage and apoptosis, abrogating systemic immunity (90). Clinically, the PACIFIC trial established the benefit of consolidation durvalumab treatment after concurrent CRT in patients with unresectable stage III NSCLC, improving both progression-free survival (≈17.2 vs. 5.6 months) and overall survival compared with the placebo; randomization occurred at 1–42 days after CRT. Exploratory analyses further suggested that earlier initiation (e.g., within ~2 weeks of completing CRT) may be associated with a greater benefit, although these findings were *post hoc* and hypothesis-generating (42, 91). Taken together, these data support PD-L1 blockade after RT/CRT, ideally without an undue delay; however, prospective trials designed specifically around the start time are needed.

Antigen-specific vaccines (e.g., sipuleucel-T) are theoretically stronger after RT, leveraging RT-induced antigen release, MHC-I upregulation, and cross-presentation to increase vaccine-primed T-cell responses. Early clinical experiences (including randomized and single-arm data from patients with mCRPC) establish feasibility and immunogenicity, with mixed but suggestive clinical readouts that merit further prospective evaluation (92, 93).

For ACT, the administration of RT first is biologically advantageous: localized irradiation induces the expression of chemokines such as CXCL16, which recruit CXCR6^+^/activated CD8^+^ T cells, increase the homing of transferred cells, and support intratumoral persistence. These results favor a “RT → ACT” sequence rather than “ACT → RT”, which is consistent with “RT-primed niches” that ACT can exploit (94).

#### Immunotherapy followed by RT

This sequence is favored for agents that debulk immunosuppression before irradiation. Among these treatments, anti-CTLA-4 has the strongest mechanism: through the Fcγ receptor-dependent depletion of intratumoral Tregs, it increases the intratumoral Teff/Treg ratio and reconditions the TME, thereby rendering tumors more permissive to subsequent RT-elicited immune clearance (95). Preclinical studies directly comparing schedules have shown that anti-CTLA-4 therapy administered prior to RT achieves better tumor control than does concurrent RT or post-RT, which is consistent with a Treg-depleting, Teff-permissive window that synergizes with hypofractionated RT (87). Emerging mechanistic data suggest that Fc-engineered anti-CTLA-4 antibodies can further reprogram the TME by inducing tumor-associated high endothelial venules (TA-HEVs), thereby facilitating T-cell trafficking and sensitizing otherwise refractory tumors to subsequent PD-1 blockade (96).

Clinical observations support this rationale. Case reports and retrospective case series of patients with metastatic melanoma have documented durable abscopal responses and survival benefits when RT is delivered during the maintenance phase of ipilimumab rather than during the induction phase. For example, patients exhibit systemic tumor regression accompanied by increased humoral and cellular immunity against tumor antigens such as NY-ESO-1 and MAGE-A3, providing immunologic correlates of the abscopal effect (97, 98). Larger retrospective analyses further suggest that compared with ipilimumab alone, ipilimumab followed by RT is associated with higher complete response rates and improved overall survival without substantially increasing toxicity (99). Together, these findings highlight that priming with anti-CTLA-4 therapy before RT leverages early immunomodulation of the TME, enabling RT to act not only as a cytotoxic modality but also as an amplifier of systemic antitumor immunity.

### Lymphocyte sparing

Lymphocytes are central to systemic antitumor immunity, and RIL has been repeatedly linked to poor outcomes across tumor types. In the immunotherapy era, preserving the lymphocyte number and function is increasingly recognized as a prerequisite for maximizing the benefit of immune checkpoint blockade and other immuno-oncology (IO) agents (100). Accordingly, RT strategies that minimize lymphotoxic exposure—both in circulating blood and to lymphoid organs—are integral to optimizing combination regimens and long-term survival.

#### Circulating blood sparing

External-beam RT unavoidably irradiates circulating lymphocytes as blood transits the treatment fields. Biophysical modeling of a standard glioblastoma plan (60 Gy in 30 fractions; 8-cm target) showed that although a single fraction exposes ~5% of circulating cells to ≥0.5 Gy, by the end of treatment, ~99% of the circulating blood pool has received ≥0.5 Gy, with a mean dose to circulating cells ≈2.2 Gy. Importantly, changing the delivery technique (IMRT vs. 3D-CRT) or dose rate has far less impact than reducing the fraction number and target volume does, underscoring the need for “immunofriendly” geometries and hypofractionation when appropriate (63).

Lymphocyte-sparing RT techniques aim to mitigate this damage. SBRT reduces the treatment volume and fraction number, decreasing the irradiation of the circulating blood pool. Studies of pancreatic cancer patients have shown that SBRT is associated with a significantly lower risk of severe RIL than conventional CRT. One month after treatment, a much smaller percentage of SBRT patients than CRT patients had severe lymphopenia (101).

Looking ahead, FLASH delivery in pencil-beam scanning proton therapy (PBS) shows promising lymphocyte sparing in dosimetric blood-flow models: single-fraction PBS-FLASH irradiated ~1.5% of peripheral blood and hypofractionated FLASH ~7.3% versus ~42.4% with conventional fractionated IMPT, translating to an estimated ~69% reduction in circulating lymphocyte depletion compared with conventional proton therapy. While clinical validation is pending, these data suggest that shorter courses, smaller fields, and ultrahigh dose rates may collectively reduce lymphotoxic exposure without compromising tumor control (102).

#### Lymphoid organ sparing

Beyond circulating blood, irradiation of lymphoid reservoirs contributes meaningfully to RIL and poor outcomes. The spleen is particularly vulnerable to RT delivered to upper abdominal fields. In pancreatic cancer CRT, a high MSD and larger splenic V10/V15/V20 were independently associated with severe post-CRT lymphopenia and shorter survival, supporting routine splenic DVH review and planning selection that limits the splenic dose when clinically permissible (103). In patients with esophageal cancer, splenic DVH parameters (V5–V30 and MSD) correlated with the nadir absolute lymphocyte count (ALC); notably, each 1 Gy increase in the MSD predicted an ~2.9% decrease in the nadir ALC, and the MSD predicted grade-4 lymphopenia during definitive CRT (104).

Bone marrow is another critical source of lymphopoiesis. With large-field pelvic or craniospinal RT, bone marrow exposure can decrease lymphocyte production. In patients with rectal cancer, the V30 of the pelvic bone marrow independently predicted a decrease in the presurgery/pre-RT ALC ratio, which in turn was associated with shorter DFS, suggesting the need for bone marrow-aware contouring and constraints during pelvic RT (105).

Finally, proton beam therapy (PBT) can reduce the integral dose to adjacent lymphoid tissues. In propensity score-matched esophageal cancer cohorts treated with definitive CRT, PBT halved the odds of grade-4 lymphopenia versus IMRT, with the clearest benefit in lower-esophageal tumors, which was consistent with greater splenic/abdominal dose sparing by protons (106). In the era of immunotherapy, protecting these lymphoid organs is essential, as they are the sources of lymphocytes for the immune response. If the lymphoid organs are damaged by RT, the ability of the body to mount an effective immune response, either on its own or in conjunction with immunotherapy, is severely compromised.

#### Lymph node sparing

Lymph nodes are key components of the immune system and are sites where immune responses are initiated and regulated. While nodal irradiation is often required for locoregional control in cancers with nodal involvement or a high risk of occult disease (107), in the immunotherapy era, preserving the structure and function of tumor-draining lymph nodes (TDLNs) has emerged as a priority whenever oncologically permissible. When combined with immunotherapy, the integrity of the lymph nodes is important for proper activation of the immune response. Importantly, our study revealed that TDLNs harbor bona fide tumor-specific memory CD8^+^ T-cell populations (Ttsm) that constitute the primary responders to PD-1/PD-L1 blockade, underscoring their pivotal role in systemic radioimmunologic synergy (108) and orchestrating a critical axis in antitumor immunity. Accordingly, RT strategies that preserve TDLNs and protect Ttsm—by minimizing elective nodal irradiation when oncologically safe and employing lymphocyte-sparing planning—are crucial to maintain the reservoir of tumor-specific memory progenitors that seed recall responses, and sustain durable systemic tumor control with checkpoint blockade.

From a planning perspective, the fundamental challenge is to balance adequate nodal control with lymphatic sparing. Compared with conventional fractionation, hypofractionated regimens can reduce the overall treatment time and the integral low-dose bath to adjacent lymphatic structures; however, their LN-specific, long-term immunologic sparing remains to be established clinically and should be presented cautiously (64). Accordingly, nodal targets should be selected and contoured according to disease site guidelines and restricted to at-risk basins whenever feasible (107).

In metastatic or oligometastatic settings, forgoing elective nodal volumes while delivering ablative RT to the primary/oligolesions has been associated with stronger immune-mediated control in preclinical models, with strategies such as sentinel LN mapping, targeted nodal management, or delayed adjuvant nodal treatment mitigating regional failure without compromising systemic immunity (109, 110).

Taken together, these data support a pragmatic approach: preserve TDLNs when safe; when nodal therapy is mandatory, tailor the fields and consider a sequence that maintains early TDLN priming and incorporate TDLN-specific constraints into planning to protect antitumor immunity.

### Targeting the TME with combined radiotherapy and immunotherapy

The TME, characterized by hypoxia, metabolic reprogramming, abnormal extracellular matrix (ECM) remodeling, increased interstitial fluid pressure (IFP), and immunosuppressive stromal and myeloid lineages, constitutes a major barrier to both RT efficacy and immune-mediated tumor rejection (111, 112). Emerging strategies targeting TME components have the potential to promote synergy between RT and immunotherapy by reversing immunosuppression, enhancing antigen presentation, and promoting immune cell infiltration (113).

#### Hypoxia-targeted radiosensitizers and immunomodulation

As previously discussed, tumor hypoxia compromises the efficacy of RT through reduced oxygen fixation by damaged DNA and simultaneously sculpts an immunosuppressive niche. Hypoxia/HIF-1 signaling promotes radioresistance, post-RT rebound programs, and immune evasion. These pathways collectively reduce antigen presentation, impair cytotoxic T-cell trafficking, and limit ICI responsiveness (114–116).

Alleviating intratumoral hypoxia can have dual benefits: radiosensitization (by restoring oxygen fixation) and immune reprogramming (by dampening HIF-1-driven immunosuppression). One prototype is a hypoxia-tropic nanozyme (Pt-HFn) that targets transferrin receptor-1 (TfR1), which is enriched in hypoxic nasopharyngeal carcinoma, and uses human heavy-chain ferritin for selective delivery. By catalyzing the degradation of H_2_O_2_ to O_2_, Pt-HFn oxygenates tumors *in situ*, increases RT-induced DNA damage, and outperforms sodium glycididazole in preclinical models, illustrating how oxygen-generating radiosensitizers can synergize with RT while potentially mitigating hypoxia-linked immune escape (117–119).

A complementary approach exploits tumor-responsive nanomaterials that penetrate hypoxic regions and amplify ICD. MMP-2-responsive WOACC nanoclusters disassemble into ultrasmall tungsten oxide nanoparticles to reach hypoxic cores; upon laser activation and RT, they generate radical species (·OH and ·O_2_^-^), increase RT-induced DNA damage, expose calreticulin, and release ATP, potentiating dendritic cell cross-priming and increasing ICI activity in orthotopic models (120). Independent platforms that reprogram hypoxic TAMs via in situ-activated nanoglycoclusters likewise increase anti-PD-1 responses, supporting a broader principle: hypoxia-addressed materials can couple radiosensitization with immune remodeling to improve the efficacy of combination therapy (121).

#### Targeting metabolic reprogramming to achieve synergy between radiotherapy and immunotherapy

Aberrant metabolic reprogramming (e.g., the Warburg effect and redox imbalance) in the TME creates a hostile niche for ROS-dependent RT efficacy and immune activation, characterized by high lactate and glutathione (GSH) levels and low H_2_O_2_ levels. Targeting these metabolic abnormalities has emerged as a promising approach to achieve synergy between RT and immunotherapy (122, 123). Within this milieu, lactate acts as an immunosuppressive metabolite that promotes M2-like macrophage polarization and Treg recruitment while curtailing the function of DCs, thereby restricting cytotoxic T-cell priming (124). Targeting these liabilities represents a path to dual radiosensitization and immune reconditioning.

Inhibiting lactate dehydrogenase (LDH) decreases intratumoral lactate levels, alleviates acidosis, and facilitates DC maturation and ICD after RT. Mechanistically, lactate supports homologous recombination (HR) DNA repair through NBS1 lactylation; LDH blockade decreases NBS1 lactylation, attenuates HR, and thereby increases sensitivity to DNA-damaging therapies. In preclinical settings, combining LDH inhibition with hypofractionated RT and PD-(L)1 blockade increased intratumoral CD8^+^ T-cell infiltration and systemic control by concurrently impairing DNA repair and reversing metabolic immunosuppression (124).

The redox imbalance characterized by high levels of GSH/low levels of H_2_O_2_ constrains ROS fixation in radiation-induced damage and limits immunogenicity. A four-in-one BAM nanohybrid (BSA-coated gold nanoclusters with MnO_2_ nanodots) exemplifies a multienzymatic strategy: GOx-like activity generates H_2_O_2_ from glucose; peroxidase-like activity converts H_2_O_2_ to ·OH; and GSH oxidase-like activity depletes GSH, amplifying lipid peroxidation and ferroptosis in synergy with RT (125). Ferroptotic death releases DAMPs such as HMGB1, promoting DC activation and CD8^+^ T-cell recruitment—an avenue to reinforce ICD and checkpoint responsiveness (126). While promising, these approaches have only been assessed in preclinical studies and warrant careful dose determination to avoid off-target oxidative injury.

Metabolic modulators also cooperate with RT by targeting innate immune pathways. RT-induced DNA damage can engage the cGAS–STING pathway, promoting type I interferon and antigen presentation; metabolic modulators and nanoplatforms should be designed to potentiate this axis without exacerbating redox toxicity, thereby enabling synergy with PD-1/PD-L1 blockade (119). Overall, metabolic interventions—reducing lactate levels, GSH depletion, and controlled ROS generation—provide versatile platforms to co-optimize RT and immunotherapy, with translation contingent on biomarker-guided patient selection and schedule-aware combinations.

#### Targeting physical TME cues to increase immune cell infiltration

Physical features of the TME, including the ECM architecture and stiffness, solid stress, and increased IFP, impede both the dose deposition/distribution of RT and leukocyte trafficking, thereby contributing to resistance to immunotherapy (127). Accordingly, remodeling rather than ablation of stromal barriers has emerged as a rational strategy to improve delivery and immune cell access.

ECM stiffness and alignment. Excess collagen crosslinking stiffens tumors and constrains T-cell migration. Inhibiting lysyl oxidase (LOX) activity or modulating the collagen topology can decrease stiffness and restore T-cell motility, improving responses to anti-PD-1 therapy in preclinical models. In desmoplastic settings such as PDAC, alignment-focused remodeling (e.g., transient disruption of LOXL2/DDR1-driven alignment) improves the penetration of therapeutics without wholesale matrix depletion, thereby facilitating subsequent cytotoxic/RT delivery and lymphocyte entry (128, 129).

Vascular/IFP normalization. The abnormal tumor vasculature increases the IFP and hinders oxygen and drug delivery. Vascular normalization strategies can transiently improve perfusion and oxygenation by increasing the effectiveness of the RT dose and enabling immune cell trafficking, provided that the dose and timing capture the normalization window (130). When integrated with immunotherapy, plans should minimize low-dose bath application, reduce the unnecessary irradiation of stromal corridors, and align the fractionation/geometry with stromal reconditioning.

### Technological innovations

Recent advances in radiation delivery are reshaping how RT is integrated with immunotherapy, moving from purely geometric precision toward spatiotemporal and immunologic design. Platforms such as proton therapy (PT), ultrahigh-dose-rate radiotherapy (FLASH-RT), and image-guided/adaptive RT can reduce low-dose exposure and collateral injury to immune-relevant structures (circulating blood, lymphoid organs, and tumor-draining nodes) while preserving the ablative dose administered to the tumor. The subsections below summarize the radiobiologic rationale, emerging clinical evidence, and pragmatic considerations for incorporating each modality into schedule-aware radioimmunotherapy.

#### Proton therapy

PT exploits the Bragg peak to deliver a highly conformal dose with a minimal exit dose, thereby decreasing integral exposure to OAR and potentially mitigating toxicity without compromising tumor control. This property is especially valuable for targets adjacent to critical neural structures (e.g., the brainstem), where careful adherence to dose constraints is essential to avoid neurological injury that could undermine survival and downstream immunotherapy delivery (131). Beyond dosimetry, accumulating clinical experience in head and neck cancer (HNC) indicates that PT can reduce acute mucosal and salivary toxicity compared with contemporary photon techniques, resulting in fewer feeding-tube placements, lower opioid requirements, and improved patient-reported quality-of-life metrics in oropharyngeal/nasopharyngeal cohorts, features that may help preserve nutritional and mucosal immune competence for combination strategies (132). In parallel, ongoing and planned trials are prospectively comparing PT with photon RT for HNC with embedded translational endpoints (e.g., immune profiling, toxicity reduction, and cost-effectiveness), reflecting the interest of researchers in this field in whether the ballistic advantages of PT can be leveraged for immuno-oncology combinations.

In thoracic oncology, the integration of concurrent chemotherapy and proton beam therapy (CPBT) followed by adjuvant ICI for inoperable stage III NSCLC has resulted in superior overall and progression-free survival compared with CPBT alone in propensity score-matched analyses, without increased toxicity; notably, grade ≥3 esophagitis was less frequent in the CPBT+IO cohort than in the CPBT alone cohort, although the mechanism remains to be clarified, and prospective validation is needed (133). These observations, together with prior evidence that PT reduces the heart/lung dose and may attenuate severe lymphopenia, support PT as a rational backbone for RT-IO strategies in the chest.

For malignant pleural mesothelioma, complex hemithoracic targets and the proximity to the heart, lung, and liver have historically limited dose escalation and combination regimens. With pencil-beam scanning PT, whole-pleural coverage can be achieved with improved OAR sparing relative to that with photons, thereby facilitating safer multimodal approaches that may include immunotherapy in selected settings (134). Across disease sites, the priority is precision: leveraging PT to spare immune-relevant tissues (e.g., lymphoid reservoirs and large vascular corridors), minimizing the low-dose bath, and maintaining treatment intensity while prospectively capturing immune-monitoring endpoints to define when and how PT best amplifies systemic antitumor immunity.

#### FLASH-RT

FLASH-RT delivers radiation at ultrahigh dose rates (typically >40 Gy/s) over subsecond timescales. Preclinical work has indicated that this delivery can widen the therapeutic window by maintaining tumor control while sparing normal tissues and has prompted interest in combining FLASH-RT with immunotherapy. Importantly, the FLASH effect depends on a constellation of beam and biological parameters (dose rate metrics, pulse structure, total dose, irradiation time, and treated volume) rather than a single threshold value, which has implications for clinical translation and immuno-oncology integration (76).

From an immune-biological standpoint, FLASH-RT can be used to reconstruct the tumor microenvironment. Across models, FLASH shifted tumor-associated macrophages toward a proinflammatory (M1-like) phenotype, with reduced Arg1/CD206 expression, increased CD86 expression, and increased CD8^+^ T-cell infiltration, features that are consistent with improved antigen presentation and T-cell priming (135). Mechanistically, recent studies have implicated a ROS–lipid oxidation–PPARγ axis: compared with standard dose-rate RT, FLASH limits ROS accumulation and oxidized LDL generation, thereby attenuating PPARγ-driven M2 polarization and sustaining macrophage support for T-cell responses (136). In medulloblastoma models, this macrophage reprogramming translated into greater CAR-T-cell infiltration and activity; combining FLASH-RT with GD2 CAR-T-cell therapy significantly improved tumor control and extended survival compared with either modality alone (137). Collectively, these findings support the concept that ultrafast dose delivery can combine normal tissue sparing with favorable immune remodeling, creating a potentially more immunopermissive platform for checkpoint blockade and cellular therapies.

Clinical development is currently underway. The FAST-01 first-in-human, nonrandomized trial established the workflow feasibility and safety of proton FLASH for the palliative treatment of painful extremity bone metastases, with pain responses comparable to those of conventional regimens and no unexpected toxicity. Building on this information, the FAST-02 protocol involved treating thoracic bone metastases using single-fraction proton FLASH, with safety/efficacy and operational metrics as primary endpoints; the results are pending (138, 139). As dose delivery and parameter standardization evolve, prospective trials incorporating immune-related endpoints will be essential to define the indications, fractionation, and sequence that maximize synergy with immunotherapy.

#### Image-guided RT and adaptive RT

Image-guided radiotherapy (IGRT) and adaptive radiotherapy (ART) improve the geometric fidelity of treatment by pairing high-quality on-board imaging with plan assessment and, when indicated, on-table reoptimization. These capabilities enable tighter margins, better OAR sparing, and the mitigation of low-dose spread, features that are increasingly relevant when RT is combined with immunotherapy. In practice, ART spans offline, online, and real-time implementations and requires robust image quality, deformable registration, dose accumulation, and integrated QA to maintain safety while adapting to inter/intrafraction anatomic changes (140, 141).

In the IGRT armamentarium, surface-guided RT (SGRT/OSI) offers nonionizing, real-time 3D surface monitoring for setup and motion management and can reduce reliance on tattoos; however, radiographic IGRT remains essential for verifying the internal anatomy, particularly for SBRT and adaptive workflows. Early online MR-IGRT/ART has shown clinical feasibility: in abdominopelvic malignancies, ~30% of fractions require online reoptimization, with a median on-table adaptation time of ~26 minutes, demonstrating a practical throughput for routine care (140–142).

CT-guided online adaptive SBRT in a PULSAR paradigm. In oligometastatic renal cell carcinoma, personalized ultrafractionated SBRT delivered with on-table CT-guided adaptation (36 Gy in 3 fractions, weeks apart) corrected daily OAR conflicts and maintained target coverage in a bulky thoraco-abdominal lesion with radiographic regression and no significant RT-related toxicity, supporting the feasibility of integrating high-dose stereotactic therapy with systemic regimens (143).

MRI-guided adaptive brachytherapy (IGABT). In patients with locally advanced cervical cancer, chemoradiotherapy followed by MRI-based IGABT achieves ~92% 5-year local control with limited severe morbidity, establishing a benchmark for adaptive, image-based dose escalation/de-escalation. In parallel, contemporary trials incorporating checkpoint blockade into definitive CRT report further improvements in the outcomes, highlighting a platform where adaptive image guidance and immunotherapy are jointly leveraged (144, 145).

MRI-guided ART (MRIgART) for ultracentral lung tumors. For targets abutting the trachea, proximal bronchial tree, and esophagus, accelerated hypofractionated MRIgART (e.g., 60 Gy/15 fx) achieved ~92% locoregional control with no grade ≥3 toxicity in an initial series; a subset of patients received concurrent systemic therapy (including immunotherapy), underscoring the potential to reduce toxicity and maintain intensity while enabling combination strategies (146).

Integration with immunotherapy. By dynamically accommodating tumor regression, organ motion, and day-to-day anatomic variability, IGRT/ART can minimize the unnecessary irradiation of immune-relevant structures (e.g., mucosa, major vascular corridors, and uninvolved lymphatic basins) and limit low-dose bath application, thereby preserving treatment delivery and potentially tempering treatment-related lymphopenia, which are prerequisites for achieving RT–immunotherapy synergy (140, 141). Prospective protocols should incorporate immune correlates (e.g., longitudinal lymphocyte counts and T-cell trafficking markers) to define the optimal sequence and adaptation triggers.

### Artificial intelligence in optimizing RT–immunotherapy combinations

Artificial intelligence (AI) has moved from proof-of-concept to pragmatic enablement in radioimmunotherapy, where the clinical benefit hinges on who receives combined therapy, how it is delivered, and when to adapt. Crucially, these tools are intended to augment, not replace, expert judgment: trustworthy deployment requires rigorous calibration, external validation, drift monitoring, and integration with quality assurance workflows. In this manner, AI becomes a systems-level catalyst for schedule-aware, immunity-preserving RT, aligning the dose distribution, delivery kinetics, and adaptation triggers with the biological windows that maximize synergy with immunotherapy.

#### Improving patient selection and treatment stratification

AI-assisted stratification can support who receives combined RT–immunotherapy and how it is delivered. For *treatment modality selection*, a large-scale “final treatment recommendation (FTR)” framework trained on registry data integrates clinicopathological factors to generate individualized survival probabilities for alternative options (e.g., surgery vs. RT). In external validation, patients whose received therapy matched the AI recommendation experienced superior survival, illustrating how such models can inform the selection of the initial modality when definitive local therapy is needed (147, 148).

With respect to response enrichment under RT–immunotherapy, radiomics can noninvasively capture tumor heterogeneity. In patients with locally advanced ESCC treated with neoadjuvant immunotherapy plus chemoradiotherapy (NICRT), a CT-based radiomics model using XGBoost predicted a pathologic complete response with AUCs of ~0.89 (training set) and ~0.80 (test set), supporting its potential to guide pretreatment enrichment and adaptive strategies (149). These findings argue for incorporating imaging biomarker endpoints into prospective NICRT/RT-IO trials to test whether radiomics-guided triage translates to a higher pCR and durable benefit across scanners and protocols.

AI also enables toxicity-aware planning. In lung cancer patients receiving ICIs followed by thoracic RT, models that combine handcrafted and deep-learning radiomic features with clinical/dosimetric variables achieved AUCs ≈0.94 for predicting symptomatic (≥G2) radiation pneumonitis (RP), substantially outperforming dosimetry-only baselines (150). Such risk models can inform dose–volume trade-offs, low-dose bath mitigation, and surveillance intensity when RT–immunotherapy regimens are designed. Prospective, multi-institutional validation and harmonized image processing (e.g., intensity standardization and scanner harmonization) are essential to ensure portability and fairness across diverse populations.

#### Optimizing radiotherapy planning and dose delivery

AI-driven innovations are strengthening precision, speed, and consistency in treatment planning and dose delivery for radioimmunotherapy while ensuring that clinical oversight remains central to decision-making. In addition to MR-only and adaptive workflows, generative models (e.g., GAN/CycleGAN) can synthesize synthetic CT (sCT) from MR data, enabling accurate dose computation while preserving the superior soft-tissue contrast of MRI and removing intermodality registration steps; these gains are contingent on rigorous HU/electron-density QA, image harmonization, and drift monitoring in online settings (151, 152).

In addition to image synthesis, deep learning-based dose predictors accelerate knowledge-based planning. U-Net families and memory-augmented variants such as MemU-Net have shown superior performance in predicting the 3D dose distribution (e.g., head and neck), improving target/OAR dosimetric surrogates versus standard baselines and supporting rapid plan iteration for schedule-aware integration with immunotherapy (153). Prospective, multisite validation and independent dose checks remain prerequisites for routine adoption.

In machine control optimization, AI spans from reinforcement learning to the direct generation of deliverable plans. Recent encoder–decoder models predict multileaf collimator (MLC) motion sequences and other machine parameters to output ready-to-treat DICOM-RT VMAT plans within seconds, achieving high gamma passing rates in retrospective prostate cohorts, thereby closing the loop from dose prediction to clinically deliverable plans and paving the way for real-time or online adaptive RT (154, 155). This paradigm reduces the dependence on repeated TPS sequencing while retaining final physics QA and physician approval.

In proton therapy, precise range control is critical when RT is combined with immunotherapy. Machine-learning-aided prompt gamma Compton imaging improves event classification and energy estimation, increasing the signal-to-background ratio and enabling more accurate distal fall-off localization (detecting millimeter-scale shifts in simulations/bench tests). Such advances support instantaneous range verification and adaptive correction, with clinical integration and workflow standardization as the next milestones (154, 156).

Overall, the use of AI in planning and delivery—sCT-enabled MR-only/ART, DL dose prediction with deliverable plan generation, and ML-enhanced proton range verification—offers a practical path to shorter planning times, tighter OAR constraints, and more reliable dose deposition, thereby protecting immune competence and improving the therapeutic index of RT–immunotherapy. Continued attention to external validation, calibration, and regulatory/ethical safeguards will determine the pace of clinical deployment.

#### Predicting and mitigating toxicity

The integration of RT with immunotherapy increases the risk of adverse events—most notably, RP and treatment-exacerbated dermatitis—making early risk stratification and on-treatment mitigation crucial. AI models that combine dosimetric metrics (e.g., MLD, V5–V30), clinicopathological factors, and radiomic features have achieved state-of-the-art performance for RP prediction in patients receiving ICIs followed by thoracic RT. In a multi-ROI framework using planning CT (GTV, PTV, and PTV–GTV), the best fusion model combining handcrafted and deep-learning radiomics with clinical/dosimetric variables yielded an AUC of ≈0.95 in the held-out test cohort, outperforming single-modal baselines and supporting pretreatment dose–volume trade-offs and surveillance intensity tailored to the risk (150). These models characterize high-risk phenotypes across tumor/target shells and the lung parenchyma rather than pinpointing a single anatomic locus and thus require prospective, multi-institutional validation with standardized image processing before routine deployment.

Beyond assessing the baseline risk, AI can assist in-course toxicity surveillance. Convolutional neural networks applied to serial on-treatment imaging (e.g., weekly CT/CBCT or MR) have shown an exploratory ability to flag early signatures of radiation-induced lung injury for timely evaluation and supportive care adjustments. In parallel, natural language processing (NLP) pipelines that parse electronic clinical notes (symptoms, timing, and negations) complement structured data capture, improving pharmacovigilance and the identification of real-world adverse events during treatment with RT–immunotherapy combinations (151, 154, 157). Harmonized lexicons, negation/temporality handling, and clinician review remain essential to ensure specificity and mitigate documentation bias.

Finally, AI-enabled adaptive workflows provide a mechanism to translate risk/monitoring signals into plan modifications. Deep-learning-based pipelines (e.g., deepPERFECT within a DAART strategy) can synthesize planning-quality data from diagnostic imaging to compress the time to treatment and facilitate earlier on-table or off-table adaptation, thereby maintaining dose constraints to at-risk structures when emerging toxicity or unfavorable anatomic changes are detected. In lung cancer, such platforms shortened the pathway from diagnosis to treatment initiation by ≥2 weeks in a virtual clinical trial and achieved noninferior plan quality after day-1 adaptation, illustrating a practical route to toxicity-aware RT delivery; prospective clinical trials with toxicity-triggered adaptation endpoints are warranted (158).

Across indications, AI is beginning to close persistent gaps in radioimmunotherapy by identifying patients most likely to benefit, transforming image synthesis and dose predictions into clinically deliverable plans within minutes, improving proton range control, and linking risk signals to actionable adaptation. The impacts of these advances ultimately depend on methodologically sound translation through prospective, multi-institutional trials; transparent reporting of discrimination and calibration; continuous performance auditing; and governance that ensures privacy, security, and fairness. In the future, hybrid mechanistic data-driven models (e.g., “digital twins” that couple dose, tumor microenvironment, and immune dynamics), federated learning to leverage diverse datasets without data pooling, and standardized decision support embedded in the RT workflow will be pivotal. If developed and validated responsibly, AI can help convert heterogeneous practices into precision radioimmunotherapy that balances efficacy with toxicity, preserves immune competence, and improves durable outcomes for patients.

## Challenges and future directions in radiation–immunotherapy combinations

The integration of RT and immunotherapy has emerged as a transformative strategy in oncology, leveraging the ability of RT to induce immunogenic cell death and systemic immune priming while harnessing immunotherapies to amplify antitumor immunity (159). However, the clinical translation and broad application of such combinations are restricted by a complex interplay of biological, technical, and clinical challenges. This section focuses on dissecting these critical barriers, which include the profound impacts of tumor and patient heterogeneity on treatment responses, the lack of reliable biomarkers for precise patient stratification, and the increased risk of synergistic toxicity requiring refined management strategies (160). By examining these challenges in depth, we aim to highlight key knowledge gaps and outline the future directions necessary to optimize the efficacy, safety, and personalization of radiation–immunotherapy combinations.

### Tumor and patient heterogeneity: barriers posed by variable tumor microenvironments and individual immune profiles

Tumor and patient heterogeneity represent critical obstacles to the effective integration of RT and immunotherapy, as they drive variable treatment responses and limit the generalizability of therapeutic strategies. This section discusses the multifaceted nature of these heterogeneities, focusing on the dynamic TME and interindividual differences in immune competence.

#### Spatial heterogeneity in the tumor microenvironment

The spatial heterogeneity of the TME imposes major constraints on the efficacy of combined RT and immunotherapy. One salient feature is the nonuniform distribution of TILs across intratumoral regions. In NSCLC, imaging mass cytometry showed that CD8^+^ T cells reside closer to tumor cells (median 41.71 μm), whereas PD-1^+^ T cells are located farther away (median 62.20 μm) and are enriched toward the tumor periphery, highlighting a spatial mismatch between radiation-induced antigen release and the availability of effector cells to execute their cytotoxic functions (161). RT may efficiently trigger antigen release in specific subregions, but the lack of effector T cells in distant areas limits the propagation of systemic antitumor immunity.

Diverse microenvironmental niches can coexist within the same tumor and are linked to treatment resistance. Even when “inflamed” areas are present and potentially sensitized to ICI, other regions remain “excluded” or “desert-like” and are characterized by sparse T-cell infiltration and persistent immunosuppression (161, 162). RT not only remodels inflamed zones but also reshapes myeloid and regulatory compartments; for example, CCR2-dependent recruitment of Tregs and monocytes has been observed after RT and is associated with resistance, underscoring the need to account for spatially divergent immune circuits (163). In parallel, coexisting vascularized versus hypoxic territories create resource gradients that both blunt radiosensitivity and restrict T-cell trafficking and effector function, thereby dampening RT–ICI synergy (161, 162).

Notably, the anatomical location of metastases introduces additional variability. In an analysis of 35 clinical reports (51 cases) published from 1973 to 2019, abscopal responses were most often observed in the lung (41%) and lymph nodes (31%) rather than the liver (15.7%) (164). Although these estimates are limited by case reports and publication biases, they align with organ-specific immune milieus: lung irradiation increases systemic CD8^+^ T-cell activation, whereas liver lesions exhibit increased MDSC infiltration, which suppresses RT-induced immune priming (165, 166).

Collectively, these spatial disparities in immune cell localization, TME subtypes, microenvironmental resources and the anatomical location of metastases undermine the synchronized actions of RT and immunotherapy, necessitating spatially tailored strategies to overcome regional resistance.

#### Interindividual variability in immune competence

Interpatient differences in systemic immune competence substantially modulate the efficacy of RT–ICI combinations. Among the readily accessible biomarkers, the ALC and the neutrophil-to-lymphocyte ratio (NLR) are consistently informative. In a pooled analysis of three phase I/II trials of metastatic NSCLC, patients with a pre-RT ALC above the cohort median (1.3 × 10^3^ cells/μL) experienced a markedly higher abscopal response rate (ARR) than those with a pre-RT ALC below the median (30.3% vs. 7.8%), underscoring the importance of preserved lymphocyte reserves before initiating RT (167).

The NLR captures the balance between protumor inflammation and antitumor immunity. In a proof-of-principle trial of RT plus GM-CSF, abscopal responders presented with a lower baseline NLR than non-responders (median of 2.29 vs. 4.24, p=0.015), suggesting that a less inflamed baseline milieu favors systemic tumor control. Independently, in a large cohort of patients with metastatic solid tumors referred for radiation oncology consultation, the baseline NLR stratified overall survival when a pragmatic cutoff point of 4 (≤4 vs. >4: 9.3 vs. 4.1 months; p<0.001) was used (168, 169). Taken together, a higher pre-RT ALC and lower baseline NLR indicate patients with superior systemic immune fitness who are more likely to benefit from RT-triggered immune activation and subsequent ICI treatment.

Therapy-related lymphopenia remains an important, modifiable barrier. Prolonged courses and/or large irradiated volumes are associated with profound and persistent decreases in the numbers of circulating lymphocytes—an adverse prognostic factor across multiple settings—highlighting the need for lymphocyte-sparing planning (e.g., minimizing elective nodal irradiation and a low-dose bath where feasible) and for monitoring ALC kinetics during treatment (170).

Host factors further shape immune competence. Aging is accompanied by an increased prevalence of Tregs within secondary lymphoid organs and a shift toward activated Treg phenotypes, with evidence of reduced clonal diversity; these changes correlate with impaired germinal center/TFH responses and suboptimal vaccine-like immunity, plausibly dampening RT-induced systemic effects (171). Obesity drives a chronic low-grade inflammatory state and myeloid reprogramming, e.g., enrichment of PD-1^+^ tumor-associated macrophages, that reshapes antitumor immunity and can modulate responsiveness to PD-1 blockade in a context-dependent manner. In parallel, IL-6, a canonical cytokine associated with obesity, exerts potent and bidirectional control over dendritic cell maturation and function, with multiple studies demonstrating IL-6-mediated constraints on effective antigen presentation and T-cell priming, mechanisms that may blunt the immunogenic consequences of RT (172, 173).

Implications. Moving beyond one-size-fits-all protocols will require the integration of baseline immune metrics (pre-RT ALC and NLR), treatment designs that preserve lymphocytes, and host-tailored immunomodulation (e.g., targeting IL-6-driven inflammation) to enhance systemic tumor recognition and durable control with RT–ICI combinations.

### Paucity of reliable biomarkers: limitations in precise patient stratification for combination therapy

The clinical success of radiation–immunotherapy hinges on the accurate identification of patients who will derive a durable benefit. However, the currently used biomarkers, including checkpoint ligand expression, immune cell phenotypes, and genomic signatures, exhibit variable predictive performance because of assay variability, marked spatiotemporal heterogeneity, and limited cross-indication validation. These constraints have hampered precision stratification and underscore the need for robust, context-aware biomarker frameworks in radioimmunotherapy.

#### Inconsistencies in immune checkpoint biomarkers

PD-L1 remains the most widely implemented biomarker for metastatic NSCLC, where high tumor cell expression (TPS ≥50%) is associated with the response to anti-PD-1 monotherapy, such as pembrolizumab; however, its predictive power is far from absolute, and many PD-L1-high tumors do not respond (174). In contrast, in the postchemoradiotherapy consolidation setting (PACIFIC paradigm), the benefit of durvalumab has been observed across most PD-L1 strata, and the ability of PD-L1 to predict a clinical benefit remains modest and uncertain—particularly when its expression is very low—illustrating the context dependence of using PD-L1 as a biomarker (175). Moreover, PD-L1 is dynamically regulated by CRT: paired pre/posttreatment specimens show the significant upregulation of tumoral PD-L1 expression and an increased stromal CD8^+^ T-cell density after cCRT, providing a pathological rationale for PD-L1 blockade but also complicating interpretations at single time points (176).

In randomized studies of SBRT plus PD-1 blockade, the greatest incremental benefit has repeatedly emerged in PD-L1-negative or otherwise “cold” tumors, which is consistent with radiation-mediated systemic immunostimulation (for example, interferon pathway induction and TCR clonal expansion in nonirradiated lesions and peripheral blood). This pattern indicates that PD-L1 expression, while correlated with anti-PD-1 monotherapy activity, poorly captures synergy with radiotherapy (74).

#### Genomic and molecular biomarkers: promise and pitfalls

The tumor mutational burden (TMB) correlates with the neoantigen load and immunotherapy sensitivity across tumor types, yet a universal cutoff (e.g., ≥10 mut/Mb) has shown limited predictive value because of cancer type-specific distributions and inconsistent associations with survival endpoints. Cancer-specific or percentile-based thresholds perform better than a single numeric bar across histologies, emphasizing the need for disease-tailored interpretation (177). In parallel, blood-based TMB (bTMB) aims to overcome tissue scarcity and sampling bias; however, prospective data (for example, B-F1RST using a ≥16/1.1 Mb cutoff) revealed nonsignificant differences in PFS at the prespecified threshold with exploratory OS separation, highlighting the need for assays and cutoff optimization and supporting multimarker approaches (178).

Notably, in radioimmunotherapy settings, immunologically cold tumors (those with a low TMB, PD-L1-null tumors, or Wnt pathway-mutant tumors) can still experience meaningful benefits after SBRT plus anti-PD-1 therapy, accompanied by systemic immune reprogramming at nonirradiated sites. These observations caution against using TMB alone to exclude patients from receiving combination strategies.

#### Challenges in biomarker validation

Emerging liquid biopsy modalities and on-therapy immune metrics hold promise for capturing dynamic responses in which static baseline markers are missed. Across five NSCLC trials using PD-L1 inhibitors, early reductions in ctDNA levels (harmonized as the maximal variant allele frequency) were consistently associated with prolonged PFS and OS, supporting the use of ctDNA as a noninvasive early indicator of a clinical benefit and a candidate endpoint for adaptive treatment algorithms (179). Complementing this approach, multiomic analyses of SBRT plus pembrolizumab demonstrate on-therapy TCR clonal expansion in both tumors and blood and interferon-driven immune activation at nonirradiated sites, which are molecular hallmarks that align with systemic (abscopal) responses. While compelling, these signatures remain associative and require prospective validation and standardization before routine deployment (74).

### Complexities of managing toxicity: synergistic adverse effects and safety considerations

The integration of RT and immunotherapy amplifies not only therapeutic efficacy but also the risk of synergistic toxicity arising from overlapping mechanisms of tissue damage and immune dysregulation. These adverse events (AEs) are complex to predict and manage, as they often involve cross-talk between radiation-induced tissue injury and immune-mediated inflammation. This section discusses the key toxicities, their underlying mechanisms, and challenges in clinical management, drawing from preclinical and clinical evidence from various tumor types.

#### Key toxicity trade-offs and implementation barriers in RT–ICI integration

Although the potential for synergy is well supported, real-world implementation is frequently constrained by toxicity and competing planning priorities. Accordingly, we summarize the major toxicity-related trade-offs that clinicians must balance when integrating RT with immune checkpoint blockade, together with practical mitigation strategies.

First, thoracic RT combined with PD-1/PD-L1 blockade can increase the risk of radiation pneumonitis in susceptible settings; mitigation relies on strict lung dose–volume constraints (e.g., V20/V5 and mean lung dose), careful sequencing, and early recognition/management of immune-mediated lung injury (180, 181).

Second, when ICIs are delivered with concurrent chemoradiotherapy, overlapping mucosal injury may amplify esophageal and gastrointestinal toxicities; supportive care, regimen selection, and conservative organ-at-risk constraints are critical (182, 183).

Third, radiation-induced lymphopenia (RIL) remains a central efficacy–toxicity trade-off because large target volumes, elective nodal irradiation, and prolonged fractionation expose circulating blood and lymphoid reservoirs; lymphocyte-sparing strategies include reducing low-dose bath (IMRT optimization, proton therapy when appropriate), minimizing unnecessary elective volumes, and shortening overall treatment time when clinically feasible (184).

Fourth, irradiating tumor-draining lymph nodes may compromise systemic T-cell priming and abscopal potential; therefore, nodal coverage should be individualized based on relapse risk, and TDLN-sparing approaches merit prospective evaluation (185).

Finally, immune-related adverse events (irAEs) can complicate RT planning by necessitating treatment holds, corticosteroids, or multidisciplinary escalation pathways; coordinated toxicity monitoring, predefined hold–resume rules, and close medical oncology collaboration are essential for safe delivery (186).

#### Mechanisms of synergistic toxicity

(i) RT-initiated inflammatory priming. Ionizing radiation causes DNA damage and diverse forms of cell death (apoptosis, necroptosis, pyroptosis, and ferroptosis), leading to the release of DAMPs, such as HMGB1, ATP, mitochondrial DNA and eCIRP. These DAMPs signal via PRRs (e.g., TLRs, RAGE, and P2X7) to activate NF-κB/AP-1 and propagate cytokine cascades, thereby exacerbating tissue injury, particularly in radiosensitive organs (lung, gut, and CNS) (187, 188). By amplifying local cytokine cascades and antigen presentation, RT creates a proinflammatory milieu that, while favorable for antitumor immunity, also increases the risk of tissue injury when combined with immune activation. In the thorax, RT-associated cytokines, including TGF-β, IL-6 and TNF-α, are correlated with pneumonitis and fibrosis risk, and the combination of anti-PD-L1 and RT can further increase TNF-α levels, potentially amplifying inflammation (189).
(ii) ICI-mediated breach of peripheral tolerance. ICIs lower activation thresholds for effector T cells and attenuate regulatory checkpoints, thereby facilitating autoimmune-like responses to normal tissues (190). In parallel, RT can expose or increase the presentation of self-antigens in normal tissues (e.g., thyroid antigens after neck irradiation), further predisposing patients to endocrine and other organ-specific toxicities, such as thyroiditis (191). Together, these processes explain the clinical entities observed in previously irradiated fields, including recall-type inflammatory syndromes, in which ICI-driven immune activation unmasks subclinical radiation-induced damage.

#### Challenges in predicting and managing toxicity

Heterogeneous risks. The combined proinflammatory effects of RT and ICIs can generate severe AEs, including pneumonitis, neurotoxicity, and radiation necrosis, but pooled clinical data do not support a uniform increase in grade ≥3 toxicity across all settings compared with ICI monotherapy (192). The risk appears to depend on the organ, modality, and regimen; for example, higher rates of treatment-associated brain necrosis have been observed in certain cohorts with brain metastasis after treatment with stereotactic radiosurgery (SRS) plus checkpoint blockade, whereas patients with thoracic tumors show variable levels of pneumonitis that are influenced by dose–volume parameters and prior RT exposure (192, 193).

Organ-specific considerations. Tolerance to RT–ICI varies by organ. In the brain, vigilance for treatment-associated brain necrosis (TABN) is warranted when combining SRS with ICIs, particularly with higher doses per fraction or large cumulative volumes (192). In thoracic disease, classic dose–volume indices (e.g., mean lung dose, V20) remain robust predictors of RP and should anchor planning when immunotherapy is integrated (193). Such organ-specific constraints highlight the need for individualized planning rather than one-size-fits-all algorithms.

Predictive modeling: promise and current limits. Radiomics and dosiomics approaches have shown potential for predicting RT–ICI toxicity (e.g., the pneumonitis risk after thoracic RT), but their performance varies across cohorts, and external validation is incomplete; prospective, multi-institutional calibration is needed before routine adoption (193).

In summary, the successful advancement of radiation–immunotherapy combinations hinges on addressing a multifaceted set of challenges that span tumor biology, patient variability, preclinical modeling, and clinical toxicity management. TME heterogeneity and interindividual differences in immune competence necessitate a shift from one-size-fits-all approaches to precision strategies, but the paucity of validated biomarkers remains a critical bottleneck for patient stratification. Concurrently, managing synergistic toxicity requires improved predictive tools and tailored mitigation strategies to balance efficacy and safety. In the future, progress will depend on integrating multiomics profiling, adaptive radiation techniques, novel biomarker discovery, advanced preclinical models, and interdisciplinary toxicity management frameworks. Overcoming these challenges will be crucial for unlocking the full potential of radiation–immunotherapy combinations, ultimately improving clinical outcomes for patients with diverse cancer types.

## Conclusions

Across preclinical and clinical studies, the success of combining RT with immunotherapy hinges on aligning “what we irradiate, when we irradiate, and how we irradiate” with the biology of antitumor immunity. Three design principles emerge. First, the sequencing schedule should be mechanistically informed, with anti-PD-1/PD-L1 drugs generally delivered after focal RT to expand reprogramming and polyfunctional CD8^+^ T cells and enable abscopal activity, whereas Treg-modulating strategies such as anti-CTLA-4 often benefit from pre-RT priming; costimulatory agonists (e.g., OX40) appear to perform best shortly after RT, when antigen presentation peaks. These patterns are consistent with the timed expansion of effector pools after RT and the loss of systemic immunity when checkpoint blockade precedes irradiation.

Second, the structure matters. Preserving the immune “substrate” is as critical as the tumor dose. Lymphocyte-sparing approaches—minimizing low-dose baths, reducing the exposure of circulating blood, and limiting doses to the spleen and bone marrow—are associated with lower rates of radiation-induced lymphopenia and better outcomes. Where oncologically permissible, sparing TDLNs should be prioritized to protect sites of priming and memory CD8^+^ T-cell differentiation, and elective nodal irradiation should be avoided in scenarios where it blunts systemic responses. Technological advances provide concrete levers: proton therapy and FLASH delivery can reduce the integral dose to lymphoid reservoirs and circulating blood, and adaptive, image-guided workflows further tighten margins and limit unnecessary exposure.

Third, substrate reprogramming is important. Rational TME modulation—hypoxia relief, metabolic rewiring, and normalization of physical barriers—can convert immune-cold niches to immune-permissive niches, amplifying the *in situ* vaccine effect of RT and enhancing responses to checkpoint blockade and cellular therapies. Together, these principles frame RT not only as a cytotoxic modality but also as a tunable immunologic adjuvant.

In the future, translation will depend on prospective, biomarker-integrated trials that (i) incorporate standardized immune endpoints (e.g., longitudinal lymphocyte counts, T-cell phenotypes, and TCR clonality); (ii) adopt lymphocyte-aware dosimetry (e.g., DVH constraints for the spleen and bone marrow and effective doses to immune cells) and prospectively test TDLN-sparing strategies; (iii) randomize schedules and sequences based on the mechanisms (post-RT PD-1/PD-L1, pre-RT CTLA-4, and post-RT costimulation); and (iv) evaluate enabling technologies (protons, FLASH, and MR-guided/adaptive RT) with immune correlations. Artificial intelligence (AI) should be positioned as a systems-level catalyst that refines patient selection, compresses planning/adaptation timelines, monitors emerging toxicity, and enforces quality assurance through calibrated, externally validated models while ensuring that clinician oversight remains central.

In summary, the curative potential of RT–immunotherapy will be realized by converging mechanism-aligned sequencing, immune-preserving precision delivery, and TME reprogramming, underpinned by AI-enabled personalization and rigorous trial design. By operationalizing these principles, we can expand the durable benefits, transform immune-cold tumors, and make abscopal-like control a repeatable clinical outcome rather than a rare exception.
